# Optimal dynamic coding by mixed-dimensionality neurons in the head-direction system of bats

**DOI:** 10.1038/s41467-018-05562-1

**Published:** 2018-09-04

**Authors:** Arseny Finkelstein, Nachum Ulanovsky, Misha Tsodyks, Johnatan Aljadeff

**Affiliations:** 1 Department of Neurobiology, Weizmann Institute of Science, 76100 Rehovot, Israel; 2 Department of Neurobiology, University of Chicago, Chicago, IL 60637 USA; 3 Present Address: Janelia Research Campus, Howard Hughes Medical Institute, Ashburn, VA 20147 USA; 4 Present Address: Department of Bioengineering, Imperial College, London, London, SW7 2AZ UK

## Abstract

Ethologically relevant stimuli are often multidimensional. In many brain systems, neurons with “pure” tuning to one stimulus dimension are found along with “conjunctive” neurons that encode several dimensions, forming an apparently redundant representation. Here we show using theoretical analysis that a mixed-dimensionality code can efficiently represent a stimulus in different behavioral regimes: encoding by conjunctive cells is more robust when the stimulus changes quickly, whereas on long timescales pure cells represent the stimulus more efficiently with fewer neurons. We tested our predictions experimentally in the bat head-direction system and found that many head-direction cells switched their tuning dynamically from pure to conjunctive representation as a function of angular velocity—confirming our theoretical prediction. More broadly, our results suggest that optimal dimensionality depends on population size and on the time available for decoding—which might explain why mixed-dimensionality representations are common in sensory, motor, and higher cognitive systems across species.

## Abstract (web summary)

Multidimensional stimuli are often represented by neurons encoding only a single dimension and those encoding multiple dimensions. Here, the authors present theoretical and experimental analyses to show that mixed representations are optimal to efficiently encode such stimuli under different behavioral modes.

## Introduction

Natural behavior requires processing of multidimensional information. For example, responding to sounds of predators or prey would depend on a neuronal representation of sound location together with acoustic features such as timber and pitch^1^, and navigation in a complex environment would require a neural encoding of one’s position and orientation in three-dimensional space^2^. Coding efficiency was suggested to be a major organizing principle in the nervous system^3,4^. Consequently, a tractable problem that has been studied extensively in theoretical neuroscience is the nature of optimal coding of a one-dimensional stimulus^5–12^. However, despite the fact that many brain regions typically integrate multidimensional information, much less attention has been given to understanding how optimal representations depend on the dimensionality of the inputs. Previous studies have suggested that stimulus dimensionality may influence the optimal tuning width^13–16^, and that neurons with mixed-selectivity tuning to multiple stimulus dimensions can simplify the readout^17^. Furthermore, modeling of short-term memory processes suggested that recall of multidimensional items depends on whether individual neurons encode one or multiple item-dimensions^18^. However, it remains unclear how the biological and behavioral constraints of the system influence the optimal dimensionality of the representation.

**Fig. 1:**
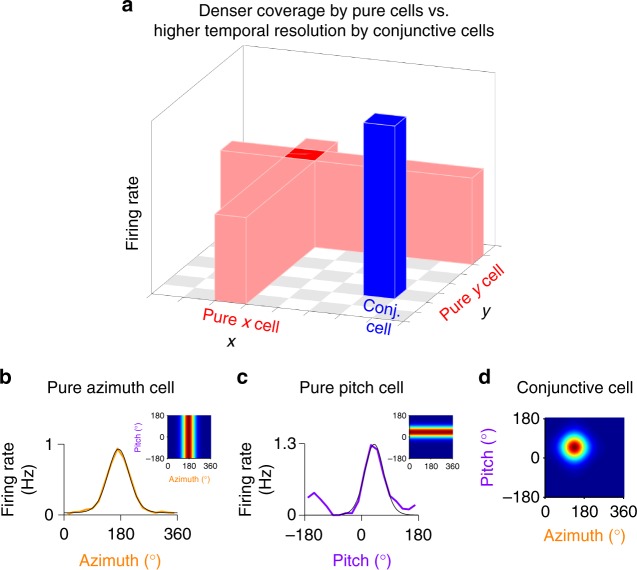
Head-direction coding by mixed-dimensionality neurons in the bat brain. **a** Schematic illustrating that a multidimensional stimulus (e.g., a 2D stimulus), can be represented with sub-populations of pure cells that are tuned to only one dimension of the stimulus, or by a population of conjunctive cells that encode the different dimensions of the stimulus jointly. Because pure cells have larger receptive fields they can tile the stimulus space more densely, compared to a population comprising the same number of conjunctive cells. Therefore, when naively considering a two-dimensional variable such as a position of a rook on a chessboard, one would expect to need only 2 × *N* pure cells (*N* cells encoding the *X* dimension and *N* cells encoding the *Y* dimension) in order to reach the same representational accuracy as *N* × *N* conjunctive cells (with *X* × *Y* tuning). However, conjunctive cells provide information about both dimensions of the stimulus at the same time, whereas decoding the activity of pure cells requires co-firing of both pure *X* and pure *Y* cells, and thus can be compromised at short decoding times. **b**, **c** Examples of 1D tuning curves of head-direction cells that we recorded in the bat dorsal presubiculum^19^: a pure azimuth cell (**b**) and a pure pitch cell (**c**), overlaid with von-Mises fits (black). Top insets in **b** and **c** illustrates schematically the directional tuning of these pure cells in the 2D space of solid angles (360° azimuth × 360° pitch). **d** An illustration of a conjunctive cell with 2D tuning to a specific combination of azimuth × pitch angles. The existence of both pure and conjunctive neurons in the same brain region suggests a mixed-dimensionality coding

A multidimensional stimulus can be represented using different strategies, since each neuron may provide information about the location of the stimulus along one or more of its coordinates. For example, decoding of a two-dimensional (2D) variable can be done using one-dimensional (1D) stripe-like cells or using 2D bump-like cells (Fig. 1a). We refer to neurons that encode a single stimulus dimension as “pure cells”, and to those that encode jointly multiple dimensions as “conjunctive cells”. Intuitively, one may expect that a population of pure cells will outperform (in terms of the magnitude of the resulting decoding error of the full multidimensional stimulus) a conjunctive cell population of the same size, because pure cells have a high firing rate in a larger fraction of the stimulus space and therefore can cover the stimulus space more densely (Fig. 1a). However, decoding the responses of pure cells will be successful only if the two pure sub-populations that represent each stimulus dimension are co-active—unlike conjunctive cells, which can provide information about both dimensions of the stimulus simultaneously, and do not depend on an effective coincidence-detection of different groups of neurons (Fig. 1a). Therefore, for fixed tuning widths, one might expect that the relative decoding accuracy of unidimensional (pure) versus multidimensional (conjunctive) codes may critically depend on two factors: the population size and the time available for decoding.

Our recent experimental finding of a multidimensional head-direction system in the bat brain, revealed the existence of neurons with pure or conjunctive representation of head-direction in azimuth and pitch^19^. Importantly, although either pure or conjunctive cells alone are sufficient to encode a two-dimensional space of solid angles (azimuth and pitch), we did observe experimentally both of these populations (Fig. 1b–d)—which together form a seemingly redundant representation. Here we first analyzed theoretically the advantages of maintaining a mixed-dimensionality representation, i.e., two populations of neurons that use either pure or conjunctive encoding schemes to represent a multidimensional stimulus. We identified several distinct regimes in terms of the optimal encoding strategy, which predicted that conjunctive cells can be advantageous for accurate decoding of a rapidly evolving stimulus over short timescales, whereas pure cells can be advantageous for long decoding times or when the neural resources are limited. We then followed up on these theoretical analyses by experimentally assessing the tuning dimensionality in bat head-direction cells during different navigation modes, and found that many cells showed a dynamical switch from pure to conjunctive tuning—in accordance with the optimal encoding strategy proposed by our theoretical analysis.

## Results

### Theoretical prediction for the decoding accuracy

We hypothesized that pure and conjunctive cells might have different relative advantages when the decoding time (*T*) is short or when the number of neurons (*N*) used for decoding is limited (Fig. 1a). Therefore, we focused on analyzing the decoding performance as function of these two variables, *T* and *N*, by first considering the decoding of a 1D stimulus. We think of *N* as the number of neurons in a particular network whose spikes are used in the decoding task. The decoding time *T* is the time-window during which the modeled neurons fire in response to a fixed stimulus being presented, and the decoding is performed using all spikes emitted during this time-window. The decoding time can be thought of as the inverse of the rate of change of the stimulus: i.e., when the stimulus changes fast, *T* becomes shorter—because the decoding should be performed faster. The magnitude of the decoding error and its dependence on the details of the model are most commonly studied by computing the Fisher information (FI) of the population responses^5,6^. The FI is a quantity that provides a lower bound for the decoding error, independently of the identity of the decoder. This is known as the Cramér–Rao (CR) bound, which is achieved in the limit of infinitely long decoding time *T* and infinitely many cells *N*^20^. However, for finite *N* and *T* the decoding error is typically larger than the bound, ${\epsilon}_{CR}$, which is equal to one divided by the square root of the FI^8^.

In Supplementary Note 1, we derived new analytical expressions for the dependence of the error on the FI before the Cramér–Rao bound is saturated. For a 1D stimulus, assuming large *N*, our theoretical analytical predictions were in excellent qualitative agreement with numerical simulations of a maximum likelihood (ML) decoder (Supplementary Fig. 1a, b; Methods). For each specific value of *N*, there was a corresponding value of *T* below which the error was significantly larger than the Cramér–Rao bound. An interesting feature of our numerical simulations is that the decoding error depends only on the FI, which here is proportional to *N* × *T*, and does not depend separately on *N* and *T*—even when the lower bound is not saturated. In other words, the decoding error from a population with, e.g., *N* = *N*_0_/2 neurons and decoding time *T* = 2*T*_0_, is the same as for a population with *N* = 2*N*_0_ and *T* = *T*_0_/2, as long as *N*_0_ and *T*_0_ are large enough. This feature was predicted by our analysis where we derived an approximate analytical expression for the error, which depends only on the FI and on the details of the tuning curve (Supplementary Note 1; and see inset in Supplementary Fig. 1a). To our knowledge, there has been no previous theoretical justification why for a population of unimodal and smooth tuning curves, deviations of the decoding error relative to the Cramér–Rao bound can be understood using the FI—as we provide here^6,8,10^.

**Fig. 2:**
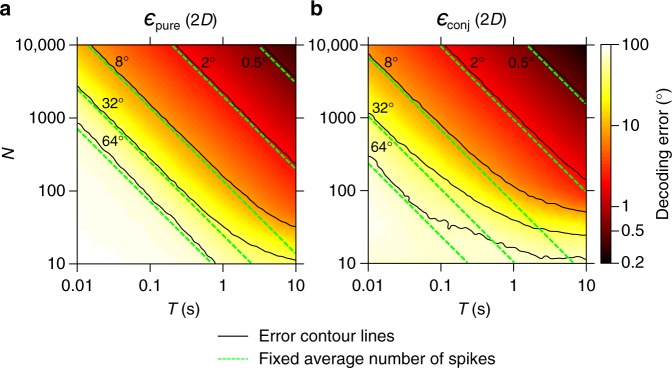
Decoding accuracy of a 2D stimulus for finite *N* and *T*: fast decoding from many neurons versus slow decoding from fewer neurons. **a**, **b** Decoding error for a 2D stimulus (head-direction in azimuth and pitch) estimated using a maximum likelihood (ML) decoder, as a function of decoding time (*T*) and the number of neurons that participate in the task (*N*). **a** ML decoder applied to the responses of a population of pure cells; **b** ML decoder applied to a population of conjunctive cells. Here the tuning curves are normalized such that the total number of spikes emitted by each population is the same (on average). The error (in degrees) is color-coded, and we also plot several contour lines (solid lines) and lines for which the total average number of spikes is fixed (dashed green lines; note the log-log scale). Deviations of the solid lines from the dashed lines represent regions in phase-space where the magnitude of the error is determined by the number of spikes as a function of *N* and *T* independently (and not only as a function of *N* × *T*). These deviations indicate that although the overall number of spikes is the same, fast decoding from many neurons differs from slow decoding from fewer neurons—and that pure and conjunctive cells perform differently under these conditions (compare **a** versus **b**)

Next, we considered the theoretical advantage of unidimensional versus multidimensional representations by analyzing the information content provided by the populations of pure and conjunctive neurons about the 2D stimulus. Decoding accuracy was measured using the scalar squared error, equal to the sum of squared errors along each stimulus dimension. In order to analyze which type of stimulus representation is optimal (in terms of the decoding accuracy), we first computed the FI analytically for both populations in the limit of large *N* and *T*. In the case of a 2D stimulus, the pure population consisted of *N*/2 cells tuned to one stimulus dimension (e.g., azimuth) and *N*/2 cells tuned to the other stimulus dimension (e.g., pitch), while the conjunctive population consisted of *N* cells tuned jointly to both stimulus dimensions (e.g., azimuth × pitch). The tuning-curve scaling (i.e., the firing rate at the preferred direction) was chosen such that each population emitted the same number of spikes on average over multiple presentations of the stimulus and multiple preferred directions (Methods). For a 2D stimulus and for our choice of relative scaling of tuning curves, the FI of the conjunctive population is equal to twice that of the pure population, because each spike of a conjunctive cell provides information about both stimulus dimensions at the same time (see derivation in Methods). Recall that the error is bounded from below by $1{\mathrm{/}}\sqrt {{\mathrm{FI}}}$ (the Cramér–Rao bound), so the factor of two difference between the FI of pure and conjunctive cell populations translates to the following ratio of their corresponding decoding errors: $\epsilon _{{\mathrm{pure}}}{\mathrm{/}}\epsilon _{{\mathrm{conj}}} = \sqrt 2$. This demonstrates that a population of conjunctive cells allows for a higher decoding accuracy than a population of pure cells, in the limit of a large number of neurons *N* and long decoding time *T* (see Fig. 2a, b: note that the solid black lines, which represent the contour lines of the decoding error, are shifted for conjunctive cells [**a**] relative to pure cells [**b**] by an amount that corresponds to dividing by a factor of $\sqrt 2$).

In contrast to our results in 1D, when we conducted a simulation in 2D (using a ML decoder, Methods), we found that the interchangeability of *N* and *T*—which is valid when *N* and *T* are large as long as their product is fixed—does not hold across the entire *N* − *T* space. Specifically, under realistic physiological conditions when *N* or *T* are not large enough^21,22^ and the error does not saturate the Cramér–Rao bound, we found that the error no longer depends on the product *N* × *T*, but rather depends separately on *N* and on *T* (Fig. 2a, b, see the divergence of the actual error contour lines [solid] from the fixed—*N* × *T* lines [dashed]). Since the average total number of spikes is proportional to *N* × *T* and is equal for the two populations, this means that given a finite number of spikes that could be emitted by a hypothetical population, the magnitude of the decoding error depends on whether these spikes are emitted by a few cells over a long period of time (small *N*, large *T*)—or by many cells over a short period of time (large *N*, small *T*). This dependence turns out to be different for pure and conjunctive cells (when *N* and *T* are small, note that the decoding errors for pure populations [Fig. 2a] versus conjunctive populations [Fig. 2b] exhibit very different divergence rates from the straight dashed lines—which represent combinations of *N*, *T* for which the total number of spikes is fixed). This suggests that the relative advantage of decoding by pure or conjunctive populations will critically depend on the number of neurons that participate in the particular computation in any specific brain system, and on the timescale of the corresponding behavior—as we will elaborate in the following sections.

### Relative accuracy of pure and conjunctive coding

**Fig. 3:**
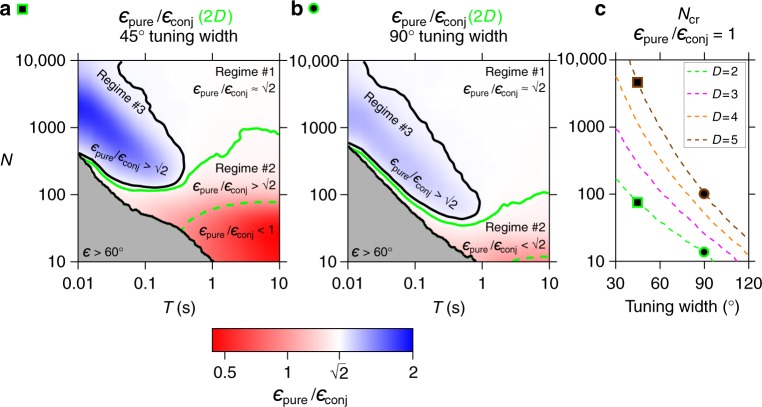
Relative coding accuracy of pure and conjunctive cells representing a multidimensional variable. **a** Relative decoding performance of pure versus conjunctive cells representing a 2D variable. We identify three regimes: regime #1, where $\epsilon _{{\mathrm{pure}}}{\mathrm{/}}\epsilon _{{\mathrm{conj}}} = \sqrt 2$, as predicted by the Fisher information (FI) calculation (white area); regime #2, where the relative performance of the pure cell population improves as compared to regime #1; and regime #3 (blue), where the conjunctive cells outperform the pure cells even beyond the FI calculation $\left( {\epsilon _{{\mathrm{pure}}}{\mathrm{/}}\epsilon _{{\mathrm{conj}}} \hskip2pt > \hskip2pt \sqrt 2 } \right)$. When the number of neurons is moderate (regime #2) we find a specific subregion below a critical value of *N* (dashed line), for which the pure cells start to outperform the conjunctive cells also in absolute terms $\left( {\epsilon _{{\mathrm{pure}}}{\mathrm{/}}\epsilon _{{\mathrm{conj}}} \hskip2pt < \hskip2pt 1} \right)$. **b** Same as (**a**) computed for neurons with a wider tuning curve (90° width at half-height). The color bar (bottom) indicates the error ratio between pure and conjunctive cells for a 2D stimulus. **c** The critical value of *N*, denoted *N*_cr_, is defined to be the population size for which the pure and conjunctive populations have the same average errors at a long decoding time, *T* = 10s (i.e., for *N* < *N*_cr_. pure cells outperform the conjunctive cells in absolute value, corresponding to the dashed lines in **a**, **b**). Shown is *N*_cr_ (*y*-axis) for different stimulus dimensions *D*, as function of the tuning width. *N*_cr_ becomes larger for narrower tuning and for higher dimensionality of the stimulus. Together, this supports the notion that in regime #2 the performance of the conjunctive population is degraded due to loss of coverage of the stimulus space by the available *N* neurons—which can happen either due to narrower tuning or due to higher dimensionality of the stimulus space. Green square and circle corresponds to the tuning widths (at 2D) for which we plot the error ratio in *N* − *T* space (**a**, **b**). Brown symbols correspond to the plots in 5D (Supplementary Fig. 2)

To assess the relative accuracy of the two types of encoding strategies by populations of pure versus conjunctive cells, we computed the ratio of the decoding errors $\epsilon _{{\mathrm{pure}}}{\mathrm{/}}\epsilon _{{\mathrm{conj}}}$ as a function of the size of the population *N* and the decoding time *T*. Using a ML decoder, we found that there are three regimes for the relative performance of the two populations (see Fig. 3a)—where the regimes depend on *N* and *T*.

Regime #1: For large *N* and large *T*, the errors of both the pure and conjunctive populations are saturated to the Cramér–Rao bound. Therefore, in this regime the conjunctive cells outperform the pure cells, such that their error ratio is $\epsilon _{{\mathrm{pure}}}{\mathrm{/}}\epsilon _{{\mathrm{conj}}} = \sqrt 2$, exactly as predicted analytically by the FI (see Fig. 3a, white region).

Regime #2: For moderate to small *N*, the relative performance of the pure cell population improves as compared to regime #1, such that the error ratio is ${\epsilon}_{\mathrm{pure}}/{\epsilon}_{\mathrm{conj}} \hskip2pt < \hskip2pt \sqrt{2}$ (see Fig. 3a, the region below the solid green line). Within that region, there is a sub-regime where the pure cell population in fact outperforms the conjunctive cell population, such that: ${\epsilon}_{{\mathrm{pure}}}{\mathrm{/}}\epsilon _{{\mathrm{conj}}} \hskip2pt < \hskip2pt 1$ (Fig. 3a, below the dashed green line). In other words, for populations smaller than a critical value of *N* (*N*_cr_), the performance of pure cells becomes absolutely better than that of conjunctive cells.

Regime #3: We also found a third regime when *T* is small. Here the conjunctive cell population outperforms the pure population by more than expected from the FI (i.e., more than in regime #1)—resulting in error ratios of $\epsilon _{{\mathrm{pure}}}{\mathrm{/}}\epsilon _{{\mathrm{conj}}} \hskip2pt > \hskip2pt \sqrt 2$ (see Fig. 3a, blue region). This suggests that as the decoding time *T* decreases, the relative advantage of the conjunctive cells over the pure cells is increasing.

As discussed in the previous section, the specific value of the error ratio that serves as the boundary between the regimes for a 2D stimulus, $\epsilon _{{\mathrm{pure}}}{\mathrm{/}}\epsilon _{{\mathrm{conj}}} = \sqrt 2$, stems from the analytically derived FI values, given our choice to scale the tuning curves such that the average population firing rate is the same for the pure and conjunctive neurons. Importantly, the proposed relative advantage of conjunctive cells for short decoding time *T*, and of pure cells for small or moderate *N*, is observed also for other scaling of the tuning curves—when the average population firing rate of pure and conjunctive cells is no longer equal (Supplementary Fig. 3; Methods).

In the simulations described so far, the spike count of each neuron was drawn independently according to its tuning curve. We also considered the case of non-zero noise correlations^23–25^, where spike counts of neurons with overlapping tuning curves are correlated (Supplementary Fig. 4a, b); cases where neurons had shared additive or multiplicative modulation of their tuning (Supplementary Fig. 4e, f); and a model in which the azimuth and pitch tuning of both pure and conjunctive cells results from shared feed-forward inputs from two hypothetical upstream populations (Supplementary Fig. 5). In these cases, we also found the same qualitative behavior: at short decoding times the error ratio is larger than at long decoding times, indicating a relative advantage for conjunctive cells (Supplementary Fig. 4c, e, f; Supplementary Fig. 5)—similar to the difference between regime #3 and regime #1 found in the absence of noise correlations, shared noise or shared inputs. Additionally, conjunctive cells became progressively worse compared to pure cells as *N* decreased (Supplementary Fig. 4d–f; Supplementary Fig. 5; Methods), similarly to what we observed in regime #2 (Fig. 3a). Taken together, this suggests that adding noise correlations, shared noise or shared inputs likely has relatively little effect on the trade-off between pure and conjunctive representations.

Further, we found the existence of the same regimes also when considering a stimulus of dimension larger than 2, with the error ratio that serves as the boundary between the regimes now being $\epsilon _{{\mathrm{pure}}}{\mathrm{/}}\epsilon _{{\mathrm{conj}}} = \sqrt D$ (where *D* is the stimulus dimensionality). For example, for a 5D stimulus, we observed that the error ratio that served as the boundary between the regimes now being $\epsilon _{{\mathrm{pure}}}{\mathrm{/}}\epsilon _{{\mathrm{conj}}} = \sqrt 5$ (Supplementary Fig. 2a). Moreover, when considering neurons with broader tuning curves, encoding either a 2D stimulus (Fig. 3b) or a 5D stimulus (Supplementary Fig. 2a), we also found the same three regimes—although the boundaries between them were different as compared to neurons with narrower tuning curves (compare Fig. 3a, b to Supplementary Fig. 2a, b). We therefore conclude that quantitatively, the exact shape and location of the regimes in the *N* − *T* space may depend on a number of factors, including: the choice of scaling, the tuning width, the assumptions about noise-structure, and the dimensionality of the stimulus. However, qualitatively, there are always three regimes where the error ratio is equal to, less than, or greater than what is predicted by the analytical FI calculation—and the existence of these three regimes is robust to the choice of model parameters. Finally, we note that changing the overall firing of the populations is equivalent (in terms of decoding) to a rescaling of time—so we expect that these results will be relevant for many brain systems operating at different ranges of firing rates and different behavioral timescales.

### Pure code is advantageous for small population size

What are the sources of performance differences of pure versus conjunctive populations for small numbers of neurons *N*, or for short decoding times *T*? In regime #2, pure cells become progressively more accurate relative to conjunctive cells, as *N* decreases—a phenomenon that can be understood intuitively through a coverage argument (see Fig. 1a): pure cells have 1D “stripe-like” regions of increased firing rate, each covering a larger portion of the stimulus space as compared to the 2D “bump-like” conjunctive cells—and hence pure cells can tile the space more effectively. Therefore, the minimal number of cells required to achieve a certain level of accuracy is expected to be smaller for the pure population than for the conjunctive one. In order to test explicitly whether the relative advantage of pure cells for small *N* stems from better coverage of the stimulus space, we analyzed the relative accuracy of pure and conjunctive cells as a function of tuning width and the dimensionality of the stimulus. We expected that if loss of coverage is the reason for the advantage of pure cells over conjunctive cells for small *N*, this effect will be more pronounced for narrower tuning curves and for higher-dimensional stimulus spaces. Indeed, we found that as the tuning width of both populations became narrower, there was a progressive increase in the critical population size *N*_cr_ below which the pure population outperformed the conjunctive population in absolute terms (Fig. 3c). We also analyzed the case for which the tuning width was different for pure and conjunctive cells, and found that the decoding accuracy depends on the relative tuning width of the two populations, in agreement with the coverage argument (Supplementary Fig. 6).

We next analyzed hypothetical stimuli of higher dimensions, and found that the relative advantage of pure cells was strongly dependent on the dimensionality of the stimulus. While for a low-dimensional stimulus space, the pure cells outperformed the conjunctive cells only for a small *N*_cr_ (Fig. 3c, see, e.g., dimensionality *D* = 2), for a high-dimensional stimulus *N*_cr_ became progressively larger (Fig. 3c, for each tuning width compare the values of the dashed lines computed for stimulus spaces of different dimensions *D*). For example, for a 5D stimulus, pure cells could outperform the conjunctive cells, in absolute terms, for a neuronal population of up to 4000 cells (Supplementary Fig. 2b, right panel: green line in inset, for 4000 neurons, is below 1 for long *T*). Taken together, these results show that at small values of *N*, the population of pure cells—i.e., neurons with low-dimensional tuning—outperforms the population of conjunctive (multidimensional) cells, because pure cells tile space more efficiently. The critical size of the network for which pure cells outperformed the conjunctive cells (*N*_cr_) increased with stimulus dimensionality and with the sharpness of the tuning curves. Therefore, for any neural system with unimodal tuning curves, we predict that if the stimulus space is high-dimensional and the neural resources are limited (small *N*)—then most cells should be tuned to a number of dimensions that is substantially smaller than the dimensionality of the full stimulus space. In other words, one should rarely find neurons that are tuned to all the dimensions of a high-dimensional stimulus space.

### Conjunctive code is more robust for short decoding time

We have described earlier that as decoding time *T* becomes shorter, the conjunctive neurons become increasingly more accurate relative to the pure neurons (Fig. 3a, b, regime #3—blue). We hypothesized that this happens because in order to decode a multidimensional stimulus from pure cells, all stimulus dimensions must be accurately estimated simultaneously, so that the decoder should effectively implement a coincidence-detection mechanism relying on separate sub-populations of low-dimensional pure cells (e.g., two sub-populations encoding pure azimuth and pure pitch: see Fig. 1a). Such a coincidence-detection mechanism is expected to fail for short decoding time *T*—as indeed observed in Fig. 3 (regime #3). This failure of coincidence-detection is expected to be ameliorated for large population size *N*. Indeed, we found that as *N* increases, there is a shortening in the decoding time *T* for which conjunctive cells are advantageous (Fig. 3a, b: note the diagonal border of the blue region).

**Fig. 4:**
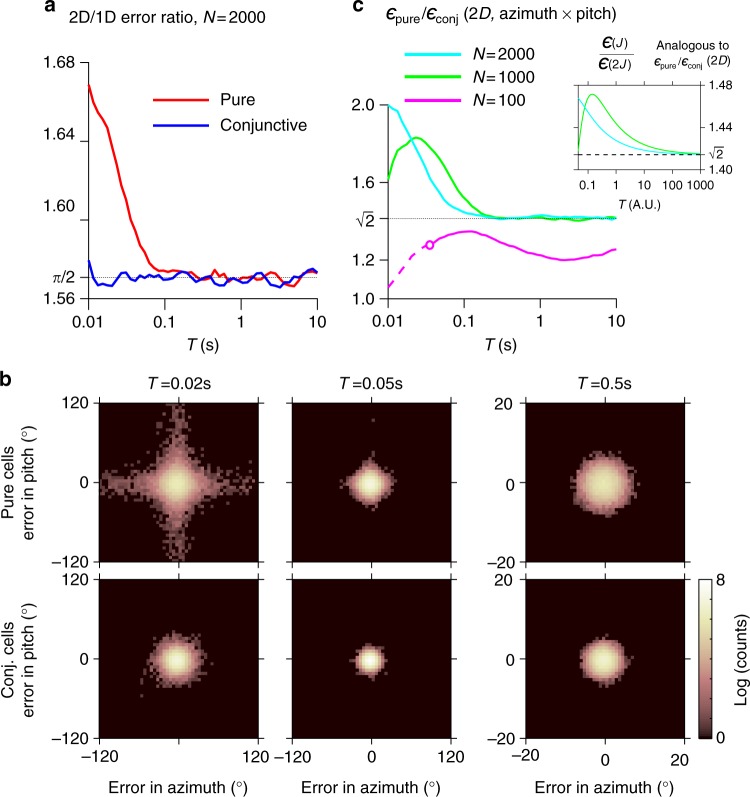
Conjunctive coding is more robust at short decoding times. **a** Error ratio in decoding 1D versus 2D stimulus for pure and conjunctive cells. At short decoding times *T*, the accuracy of pure cells in decoding of a 2D stimulus drops as compared to a 1D stimulus (red line: 2D/1D error ratio increases at short *T*)—whereas for conjunctive cells this ratio is independent of *T* (blue line: relatively flat). **b** Distribution of the decoding error in azimuth and pitch for pure cells (top) and conjunctive cells (bottom), for different decoding times *T* (columns). At short *T* (left column), pure cells have larger spread in the 1D error magnitude for azimuth or pitch, which may compromise the estimation of the combined 2D stimulus. As *T* increases, the error variance of pure cells becomes symmetric (circular) in both dimensions, resulting in a more accurate combined estimate. For the conjunctive cells, the variance is symmetric in both dimensions for all decoding times (compare top row to bottom row). **c** Ratio of the decoding error of a 2D stimulus (azimuth × pitch) by pure versus conjunctive cells, as a function of *T*. The ratio is plotted for three different population sizes (*N*). For short *T*, pure cells fail to integrate the two dimensions of the stimulus (failure of coincidence-detection)—and therefore the relative decoding performance of conjunctive cells improves as *T* gets shorter. At longer *T*, the relative decoding performance by pure and conjunctive cells converges to a fixed ratio, and as *N* increases this ratio asymptotically approaches the ratio of $\sqrt 2$ predicted by the Cramér–Rao bound (dashed black line). Inset: the ratio of the pure and conjunctive decoding errors $\epsilon _{{\mathrm{pure}}}{\mathrm{/}}\epsilon _{{\mathrm{conj}}}$ can be estimated from the theory by dividing the error for a given value of the FI by the error for twice that value of the FI (see Supplementary Note 1). The predicted ratio exceeds $\sqrt 2$ for small *T*, similar to the simulation results, but fails to capture the differences in the maximum value of the ratio for different values of *N* (*N* = 1000 and *N* = 2000)

According to the coincidence-detection hypothesis, the relative advantage of conjunctive cells for small *T* is expected to occur only when decoding the stimulus value along multiple dimensions simultaneously (e.g., 2D azimuth × pitch), but not when decoding each dimension separately (e.g., 1D azimuth or pitch). To test this, we considered an example 2D stimulus—a point in the two-dimensional space of solid angles (azimuth and pitch); we then computed the decoding errors for azimuth or pitch (1D) separately, and for azimuth × pitch (2D) jointly—for both pure and conjunctive cells. To allow direct comparison of the pure and conjunctive populations, we compared the ratio of the decoding errors for 2D/1D. We found that this ratio was fixed for conjunctive cells (Fig. 4a, blue), whereas for pure cells this ratio diverged from a constant value and became larger as *T* decreased (Fig. 4a, red). These results indicate that the disadvantage of pure cells for short *T* stems from a failure to integrate the different dimensions of the stimulus into a multidimensional representation— because coincidence-detection across multiple dimensions fails for short *T*.

To understand further how the estimation accuracy of the multidimensional stimulus depends on the decoding time, we analyzed the decoding-error distribution across the stimulus space (360° azimuth × 360° pitch) for pure and conjunctive cells, at different decoding times *T* (Fig. 4b). As expected, as *T* increased, the error became smaller for both types of cells (Fig. 4b, see the decrease in spread of the error distribution when going from left to right; note that the right-most plot corresponding to *T* = 0.5s has a different scale [zoom-in]). Importantly, for pure cells (top row), the error magnitude along each dimension had a large spread (note the “cross-shaped” distribution in Fig. 4b, top left)—meaning a poor combined estimate of the 2D stimulus when the decoding of either the azimuth or the pitch sub-populations fails. By contrast, for conjunctive cells, the error magnitude in azimuth and pitch had small spread, manifested in a circularly symmetric error distribution with only few extremely poor estimates (Fig. 4b, bottom left). As *T* increased, the marginal 1D error distribution for pure cells became progressively narrower (Fig. 4b, top right), and eventually the shape of the 2D error distribution for pure cells became similar to that observed for conjunctive cells (Fig. 4b, bottom right).

We further analyzed how the failure of pure cells at short decoding time *T* was related to the overall number of spikes emitted by each of the populations. We found that the transition from regime #3 to regime #1 occurs once *N* and *T* are such that there are no longer instances when one of the sub-populations of pure cells fires below a certain critical number of spikes required for accurately estimating of both stimulus components simultaneously (akin to coincidence-detection, see Supplementary Fig. 7). In other words, the advantage of conjunctive cells is manifested when one of the sub-populations of pure cells has a non-negligible probability to emit too few spikes, resulting in a very poor estimate of the stimulus along at least one dimension.

Finally, the theoretical estimate obtained for the 1D error (Supplementary Note 1, and Supplementary Fig. 1a) also provides a qualitative prediction for regime #3. We find that, like the ratio computed from simulations, this theoretical estimate exceeds $\sqrt 2$ for short decoding times (see Fig. 4c inset), which can be understood intuitively by noting that when the error is not saturated to the Cramér–Rao bound ($\epsilon {\mathrm{/}}\epsilon _{{\mathrm{CR}}} \hskip2pt > \hskip2pt 1$ in the inset to Supplementary Fig. 1a), increasing the FI by a factor of 2 results in reduction of the error by a factor greater than $\sqrt 2$.

Taken together, our analyses indicate that conjunctive cells have an advantage over pure cells in decoding a multidimensional stimulus at short decoding times *T*. We showed that this occurs because conjunctive cells can represent all stimulus dimensions at the same time, whereas decoding by pure cells relies on coincidence-detection of different stimulus dimensions by different groups of cells—a mechanism that fails for short decoding times.

### Decoding from mixed-dimensionality populations reveals synergy

We next analyzed whether conjunctive cells can improve the performance of pure cells in a synergistic manner. To reveal such a putative synergistic effect we normalized the absolute error of the mixed population $\epsilon _{{\mathrm{mix}}}$ (Supplementary Fig. 8a) by $\epsilon _{{\mathrm{mix,independent}}}$, the error expected under the null assumption that the different sub-populations contribute independently towards improving the decoding accuracy, without interactions (observing a ratio <1 would then indicate a synergistic interaction; Methods). We found that for short decoding times the normalized error $\epsilon _{{\mathrm{mix}}}{\mathrm{/}}\epsilon _{{\mathrm{mix,independent}}}$ was lowest when 50–80% of the cells in the mixed population had pure tuning, and the rest were conjunctive (Supplementary Fig. 8b, green square). This stemmed from the fact that the addition of conjunctive cells reduced the chance of catastrophic decoding errors by pure cells at short decoding times (Supplementary Fig. 8c, note the cross-shaped error distribution for the pure-only case [top], but not for the mixed case [middle]). We therefore conclude that pure cell become less error-prone at short decoding times *T* when they are mixed with a population of conjunctive cells.

### Two behavioral modes in bats with different temporal scales

**Fig. 5:**
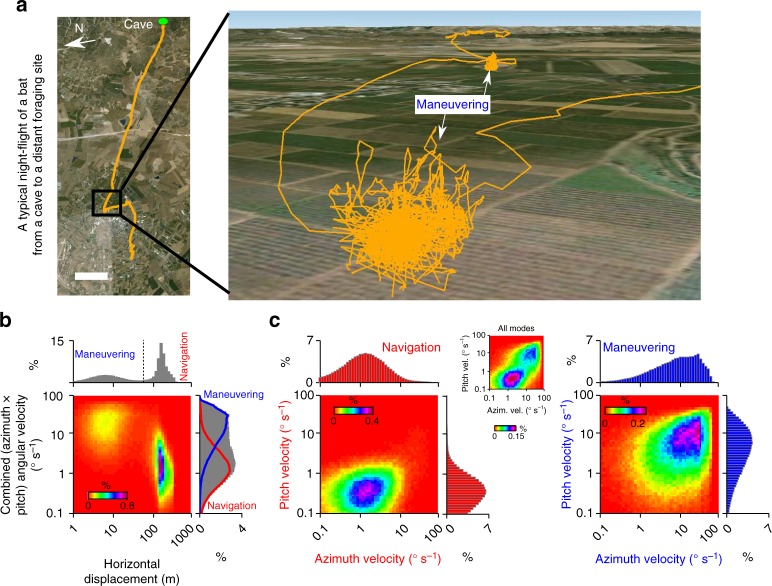
Bat orientation behavior consists of two distinct modes—navigation and maneuvering—that are characterized by different angular speeds. **a** Left, a typical night-flight of an Egyptian fruit bat from its roosting cave (green ellipse) to distal foraging sites; scale bar, 2 km. Right, a zoom-in on the natural orientation behavior, showing epochs of navigation (commuting)—characterized by relatively straight flights during which there was little modulation of heading-direction, interspersed by periods of intensive maneuvering around fruit-trees (indicated by white arrows)—characterized by rapid turns (imagery produced using desktop version of Google Earth Pro). **b** Distribution of the combined (azimuth × pitch) angular velocity versus horizontal displacement of the bat. Maneuvering mode and navigation mode were classified according to the threshold (vertical dashed black line) at the minimum of the marginal distribution of the horizontal displacement. The marginal distribution of combined angular velocity is shown for all data (grey), and separately for navigation (red) and maneuvering modes (blue). **c** Angular-velocity distribution computed separately for pitch versus azimuth during navigation (left) and maneuvering (right). Inset (middle) shows the angular velocity distribution for the entire session. Note that during maneuvering (right), angular velocities in azimuth and pitch were correlated, suggesting that bats’ maneuvers were composed of rapid rotations in both azimuth and pitch. The data in **b** and **c** were pooled over all bats and flights that we recorded (45 bats with one nightly flight for each bat^26^)

In the previous sections, we showed that the relative decoding accuracy of pure versus conjunctive cells depends on the decoding time. While it is not trivial to estimate the decoding time at which a realistic biological network is operating, there is a closely related and experimentally tractable timescale—namely, the timescale over which a behaviorally relevant stimulus is changing. We therefore analyzed the statistics of change in heading-direction of Egyptian fruit bats during natural navigation outdoors, using data that was previously collected using miniature high-resolution GPS-devices^26^ (Methods). In a typical nightly flight, individual bats traverse distances of up to 25 km from the roosting cave to a distant foraging site (Fig. 5a, left). A closer examination revealed that rather straight commuting flights were often interleaved with epochs of intense maneuvering, which correspond to foraging for fruits around fruit-trees (Fig. 5a, right). This suggested the existence of two behavioral modes: a navigational mode, consisting of long straight flights with little directional modulation—and a maneuvering mode, with rapid changes in heading-direction (the two modes are shown in Supplementary Movie 1).

To further test for the existence of two distinct behavioral modes, we computed the combined angular velocity of the bat in azimuth and pitch, and plotted it against the horizontal displacement—a parameter that measures the Euclidean distance traversed by the bat (Fig. 5b, see Methods). This analysis revealed two very distinct behavioral clusters—one that was characterized by large horizontal displacement and small angular velocity (corresponding to long-distance navigation), and the other with small horizontal displacement and large angular velocity (corresponding to maneuvering). This pronounced separation allowed to analyze each behavioral mode independently (Fig. 5c: navigation—left, and maneuvering—right). There was a positive correlation between azimuth and pitch velocities in both modes (Fig. 5c, inset)—and during maneuvering in particular, high velocity in azimuth co-occurred with high velocity in pitch (Fig. 5c, right; Pearson correlation coefficient *r* = 0.26, *P* < 0.001). This suggests that rapid changes in heading-direction angle during maneuvering are not restricted to only azimuth or pitch—raising the need for simultaneous encoding of both of these dimensions.

### Dynamic tuning of bat head-direction cells matches theory

Can the advantages of different encoding strategies, as highlighted by our theoretical analysis, be mapped onto the modes of bats’ natural orientation behavior? During long-distance navigation, the available decoding time can be relatively long, because in this mode the bats fly rather straight and therefore change their heading-direction very slowly. Thus, for low angular velocity (long *T*), pure cells are not expected to be prone to errors due to insufficient decoding time, and therefore may perform the task efficiently with relatively small number of neurons and without increasing the firing rates or recruiting conjunctive neurons. By contrast, during maneuvering the bat turns frequently with rapid modulations of heading-direction in both azimuth and pitch. Therefore to maintain a comparable decoding accuracy at high angular velocity (short *T*), we postulated based on our theoretical analysis that the head-direction system could exhibit some of the following dynamics during maneuvering: (i) an increase in the firing rate of pure cells; (ii) recruitment of additional cells (i.e., to increase *N*, see Fig. 2a); and (iii) a shift from a pure to a conjunctive representation.

**Fig. 6:**
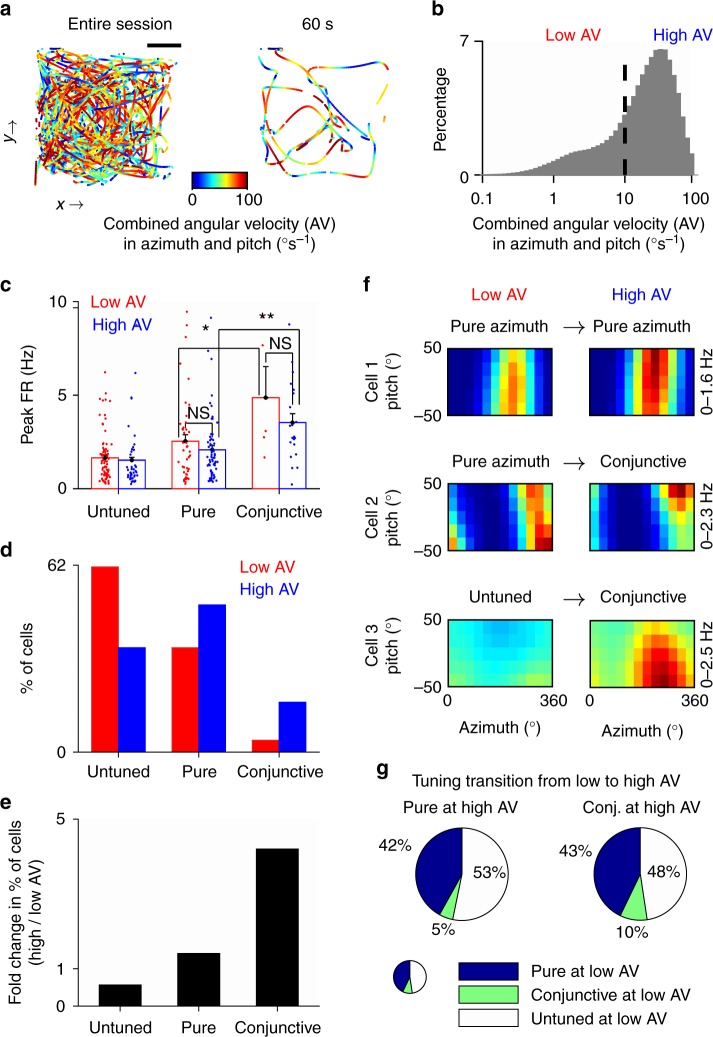
Dynamic shifts from pure to conjunctive tuning in head-direction cells as a function of angular velocity. **a** Top view of the trajectory of a bat crawling on the floor of a horizontal arena, color-coded according to combined (azimuth × pitch) angular velocity (AV). Left, trajectory from an entire session; scale bar, 10 cm. Right, 60 s trajectory from the same session. During crawling, there were frequent transitions between epochs of low and high angular velocity. **b** Distribution of the combined (azimuth × pitch) angular velocity in crawling bats. A cutoff of 10 degrees per second (dashed line) was used to separate between low and high angular velocity. **c** Peak-firing rates of untuned, pure, and conjunctive cells during low or high angular velocity. There was no significant difference in the peak-firing rate computed at different angular velocities. For both low and high angular velocity, conjunctive cells had higher peak-firing rate than pure cells. Error bars, mean ± s.e.m.; ^*^P < 0.05, ^**^P < 0.01, using Student’s *t*-test. **d** Fractions of untuned cells and cells with pure or conjunctive tuning to azimuth and pitch, plotted separately for low (red) versus high angular velocity (blue). **e** Ratio between the percentages of cells in high/low angular velocity, plotted separately for each of the 3 cell classes (3 bars). The percentage of conjunctive cells increased fourfold in high versus low angular velocities. **f** Examples of 3 neurons recorded in the dorsal presubiculum of crawling bats with different tuning properties. Shown are 2D rate-maps as a function of azimuth and pitch, computed separately for low (left) and high angular velocity (right). The significant dimensions to which each cell was tuned, under low or high angular velocities, is indicated above the map. Color scale: zero (blue) to maximal firing rate (red), values in Hz are indicated. **g** Proportions of tuning-type transitions between low and high angular velocities, for cells with pure (left) or conjunctive tuning (right). For example, the right pie chart shows—for cells with conjunctive tuning at high angular velocity—what percentage of these cells had pure, conjunctive, or no tuning at low angular velocity

To test these predictions experimentally, we turned to recordings of head-direction cells in the dorsal presubiculum of bats that were freely crawling in the laboratory, using new analysis of data reported in Finkelstein et al.^19^ (Methods). We reasoned that the optimality principles that might have emerged in the head-direction system in order to support different modes of natural orientation behavior could be reflected in the circuit dynamics also under laboratory conditions. During crawling, bats also exhibited epochs of both slow and fast turns of the head (Fig. 6a), with combined angular velocity of the head in azimuth and pitch spanning a similar range to the range that we measured during orientation behavior in the wild, albeit with somewhat different statistics (Fig. 6b, compare with the marginal distribution in Fig. 5b—right marginal histogram). This allowed us to separate the crawling behavior into two parts, based on low versus high angular velocity in azimuth and pitch (Fig. 6b, dashed line)—by applying the same threshold value that distinguished navigation and maneuvering modes in the wild.

We next compared the head-direction tuning in azimuth and pitch for low versus high angular-velocity conditions. First, we did not find a significant change in the firing rates between low and high angular velocities (Fig. 6c, compare blue and red bars within each group of neurons—conjunctive, pure, untuned). Second, we found a recruitment of both pure and conjunctive cells at high angular velocity from the pool of directionally untuned cells (Fig. 6d). Third, the proportion of pure azimuth and pure pitch cells increased only moderately at high angular velocity, whereas the recruitment of conjunctive cells was more prominent (Fig. 6e). In fact, at high angular velocity, 16.5% of cells exhibited conjunctive tuning to azimuth and pitch—4 times more than for low angular velocity (Fig. 6e), consistent with our theoretical predictions.

Next, we examined in more detail to what extent the dynamic changes in head-direction tuning—and in particular, the increase in the fraction of conjunctive cells (Fig. 6e)—resulted from recruitment of directionally untuned cells, as compared with transitions from a pure to a conjunctive representation. We found that 42% of the cells with pure tuning at high angular velocity had the same tuning at low angular velocity (Fig. 6f, example cell 1 had a pure azimuth tuning under both conditions)—whereas 53% were untuned at low angular velocity (Fig. 6g, left). By contrast, only 10% of the cells with conjunctive tuning at high angular velocity were also conjunctive during epochs of low angular velocity, whereas 47% developed a conjunctive tuning from an untuned state (Fig. 6f, example cell 3 and Fig. 6g, right). Importantly, we found that the remaining 43% of the conjunctive cells had pure tuning to either azimuth or pitch at low angular velocity (Fig. 6g, right). These cells gained additional tuning to the other angular dimension at high angular velocity (Fig. 6f, example cell 2)—thus dynamically switching from pure to conjunctive representation. We verified our findings by analysing the data over multiple ranges of angular velocity, and observed that that the proportion of conjunctive cells indeed increased with angular velocity, a process that was accompanied by gradual narrowing of the tuning in azimuth or pitch (Supplementary Fig. 9). Taken together, this demonstrates that tuning dimensionality of head-direction cells is not a fixed property, but can switch dynamically as a function of angular velocity—consistent with the proposed improvement to the population code accuracy that was suggested by our theoretical analysis.

In summary, our theoretical and experimental analyses suggest that mixed-dimensionality representations by pure and conjunctive cells are not redundant, but in fact can outperform an encoding strategy that relies on only one of these cell types—by matching dynamically the neuronal population size and type to the behavioral task at hand.

## Discussion

Multidimensional variables can be represented in the nervous system using neural tuning curves with different shapes and dimensionalities. In the case of the head-direction system of bats, our previous experimental work has suggested the existence of a mixed-dimensionality coding by both pure and conjunctive neurons tuned to head azimuth and pitch^19^. Here we used theoretical, computational, and experimental approaches to investigate the advantages of such a mixed-dimensionality representation, by considering a biologically relevant situation in which the number of active neurons and the decoding time might change dynamically.

Our theoretical analysis demonstrated that fast decoding from many neurons is not equivalent to slow decoding from fewer neurons, and the optimal performance depends in fact on whether pure or conjunctive cells are used for decoding. At long timescales, a population of pure cells can be more efficient in representing a high-dimensional stimulus using fewer active neurons. We found that the critical population size at which pure cells can outperform conjunctive cells increases with the dimensionality of the stimulus. We demonstrated that the critical value can be on the order of 1000 to 10,000 neurons for 4–5 stimulus dimensions. For comparison, the size of a single barrel in the somatosensory cortex of mice (involved in complex dynamic computation of whisker kinematics in 4–5 dimensions) is about 5000 neurons^27^. Many neurons in the head-direction system are also likely highly multidimensional, and encode other variables in addition to head azimuth and pitch^19,28,29^. Furthermore, in systems where neurons fire sparsely, the number of neurons needed for appropriate coverage of the stimulus space can be significantly higher^30^. This suggests that, when decoding high-dimensional signals, an accurate conjunctive code could require more neurons than the brain typically dedicates to a particular decoding problem—making a pure code favorable in such scenarios. By contrast, conjunctive cells can provide a more robust decoding for a rapidly changing stimulus, when only a short decoding time is available—but this will require more active neurons.

Our theoretical results therefore predicted that if the rate of change of a multidimensional stimulus will increase (e.g., during vigorous maneuvering or faster movement), the system will perform optimally by recruiting more neurons to the task, with a preference for recruiting conjunctive cells—a prediction that was confirmed by our experimental findings in the head-direction system of bats (Fig. 6). This prediction is also supported by recent findings from entorhinal-cortex recordings in mice^31^. We therefore proposed here a novel role for conjunctive neurons as a neural substrate for encoding behaviorally relevant variables at fine temporal scales—although we note that conjunctive coding also likely has other important functions, such as representing multidimensional information in complex cognitive and working-memory tasks^18,32,33^.

An important question is how the conjunctive representation formed? One possibility is a feed-forward network in which conjunctive cells are formed by inputs from pure cells. Such an architecture, which leads to formation of a conjunctive representation from functionally distinct dendritic inputs, was reported for hippocampal place cells^34^. In this scenario, conjunctive cells may inherit the unreliability of pure cells at short decoding times. However, we showed that at short decoding times, the robustness of pure coding can be improved by increasing the number of neurons participating in the task. Therefore, if in the case of head-direction cells the conjunctive neurons in the dorsal presubiculum receive converging inputs from a sufficiently large population of pure cells located in subcortical areas^2,29,35^, the resulting conjunctive representation will be immune to failures at short decoding times.

To show a feasibility of this architecture we modeled a feed-forward network in which downstream pure or conjunctive cells were constructed by pooling from two large upstream populations of pure cells. We showed that decoding from downstream conjunctive cells was more accurate at short decoding time compared to decoding from downstream pure cells, even though both types of cells were constructed from the same upstream populations (Supplementary Fig. 5). This suggests that if anatomical constraints preclude readout from a large number of upstream pure cells then, at short decoding times, it would be advantageous to decode from an intermediate layer composed of conjunctive cells—as compared to readout from an intermediate layer of pure cells of the same size. It is important to emphasize that our claim is not that conjunctive neurons are able to know more about the stimulus than is known by their inputs. Rather, if the stimulus information must be forced through an anatomical “bottleneck” of *N* neurons, then doing so via a pure or via a conjunctive population will affect the amount by which the decoding accuracy is reduced—in a way that depends also on the decoding time *T*.

An alternative way to construct conjunctive head-direction tuning is through an attractor network where the conjunctive representation is not formed hierarchically from pure cells but rather emerges from recurrent connectivity. Rubin et al.^36^ have shown that a mixed-dimensionality representation of head-direction similar to the one seen experimentally can be maintained by an attractor network. Moreover, their theoretical analysis demonstrated that the fraction of pure versus conjunctive neurons could be modulated dynamically, suggesting the possibility that an external signal (e.g., angular velocity) could shift the head-direction system towards the encoding scheme that is optimal for the current behavioral mode—as we found experimentally here.

We expect that our approach could help identify optimal population codes in other encoding-decoding paradigms, beyond the head-direction system and the unimodal tuning that we considered here. In the mammalian navigation circuit, conjunctive coding was reported in the hippocampus for azimuthal head-direction and place tuning^37–39^; for place, goal-direction, and goal-distance tuning^40^; and for various combinations of azimuthal head-direction, place, grid, and border tuning—in the presubiculum, parasubiculum, and entorhinal cortex^28,41–43^. Notably, in addition to conjunctive representations, all these regions contain also neurons with pure tuning to the same parameters (e.g., pure tuning to grid or to head-direction)—suggesting that mixed-dimensionality coding exists for various multidimensional stimuli beyond those encoding circular variables.

Mixed-dimensionality representations also exist beyond the navigational circuitry, with evidence for a mixture of both pure and conjunctive coding in different sensory areas: For example, visual feature selectivity in the salamander retina^44^ and in primate visual cortex^45^; somatosensory neurons tuned to different kinematic features of the whisker motion and touch in the rodent somatosensory pathway^46,47^; neurons with different auditory feature selectivity in auditory field *L* of birds^48^, and in the primary auditory cortex of cats and ferrets^49,50^. Neurons in the ferret auditory cortex were found to represent pitch, timbre, and spatial location of the sound conjunctively^1^, but there was also a dynamic multiplexing of these features, so that different dimensions of the stimulus could be represented independently within specific time-windows following sound presentation. Pure and conjunctive representations were also found in the midbrain of weakly electric fish, where neurons were reported to respond to single or multiple electrosensory features^51^.

Beyond classical sensory regions, mixed-dimensionality tuning was also found in the context of multisensory representations^52^, including multisensory tuning to optic flow and vestibular inputs^53^ or optic flow and locomotion^54^. Furthermore, neurons with mixed-dimensionality coding for hand position and velocity were found in the motor cortex^55^, and neurons with mixed-dimensionality coding for 3D head motion were reported in the motor subdivision of the superior colliculus^56^. Finally, neurons with mixed-dimensionality coding were found in face-processing areas in monkeys—where cells were reported to encode up to eight dimensions in the face feature space, in either pure or conjunctive fashion^57^. As noted above (see Fig. 3c), the tradeoffs in encoding by pure and conjunctive population codes, which we found here, will be even more prominent in such an 8-dimensional space.

Taken together, our analysis proposes a new role for a mixed-dimensionality encoding strategy by pure and conjunctive populations, with respect to the time available for decoding, the number of neurons involved in the task representation, and the dimensionality of the encoded stimulus. Using the bat head-direction system as an example, we demonstrated that neuronal circuits can switch dynamically from pure to conjunctive representations for different behavioral modes, in line with the optimality principles revealed by our theoretical analysis. We expect that these principles can be generalized to other neuronal systems that encode multidimensional representations—such as sensory, motor, and higher cognitive areas—suggesting a new fundamental link between natural behaviors and neural computation.

## Methods

### Animals

This study includes new data analysis of previously published experiments^19,26^ conducted on Egyptian fruit bats, *Rousettus aegyptiacus*. All experimental procedures were approved by the Institutional Animal Care and Use Committee of the Weizmann Institute of Science, and are detailed in refs.^19,26^

### Tuning curve fitting and model construction

To investigate the relative advantage of pure versus conjunctive representations in the brain, we focused on the example of the head-direction system in the bat dorsal presubiculum (a part of the hippocampal formation), which was recently shown to contain populations of neurons employing both strategies—pure cells (tuned to either azimuth or pitch: Fig. 1b, c) and conjunctive cells (tuned jointly to azimuth × pitch: Fig. 1d)^19^.

We fitted the one-dimensional head-direction tuning curves of neurons (see Fig. 1c, d) with a circular normal function, known also as von-Mises function—which has the following form:

1 $$R_i(\varphi ) = c_1e^{\kappa {\kern 1pt} {\mathrm{cos}}\left( {\varphi - \varphi _i} \right)} + c_2.$$

Here *φ*_*i*_ is the preferred direction of the cell in radians, *φ* is the stimulus value according to which the firing rate is determined, and *κ*, *c*_1_, and *c*_2_ are constants corresponding to the tuning width, peak-firing rate, and baseline firing rate, respectively.

For 2D tuning, we fitted the 1D azimuth (*φ*) and 1D pitch (*θ*) tuning curves of conjunctive neurons with one-dimensional von-Mises functions, and combined these to give a two-dimensional von-Mises function (see Fig. 1e):

2 $$R_i(\varphi ,\theta ) = c_3e^{\kappa _1{\kern 1pt} {\mathrm{cos}}(\varphi - \varphi _i) \,+\, \kappa _2{\kern 1pt} {\mathrm{cos}}(\theta - \theta _i)} + c_4,$$

where *κ*_1_, *κ*_2_ control the tuning widths in the azimuth and pitch directions, and *c*_3_ and *c*_4_ are constants corresponding to the peak-firing rate and baseline firing rate, respectively.

The model we constructed for the neural responses consisted of two sub-populations (pure and conjunctive) described by Eqs. (1), (2), respectively. The pure population consisted of *N*/2 cells tuned to azimuth and *N*/2 cells tuned to pitch, while the conjunctive population consisted of *N* cells tuned jointly to azimuth × pitch. All preferred head-directions were drawn randomly from a uniform distribution between 0 and 2*π* in azimuth and pitch.

Choosing pitch tuning to span the entire 360° range is in-line with the experimental finding of a toroidal coordinate system whereby azimuth and pitch tuning are coded independently^19^. Specifically, we have shown^19^ that both azimuth and pitch are encoded as circular variables (0–360°). When bats were crawling on a horizontal arena, head-direction angles covered the full range of azimuth (0–360°), but had a more limited range of pitch (approximately ±45° pitch). To demonstrate the circular tuning to pitch we recorded head-direction cells while bats traversed on the inside of a vertically positioned ring that allowed sampling the entire range (0–360°) of pitch angles. We showed that during crawling on a horizontal surface (where the pitch range was limited), pitch tuning curves were a “clipped version” of the full tuning to pitch that was observed when the entire pitch range was sampled on the vertical ring. Thus, in our model the tuning curves of individual neurons spanned the entire range of azimuth and pitch (i.e., both dimensions were circular 0–360°). In order to compare tuning width and firing rate of pure and conjunctive cells for the entire range of azimuth and pitch, we used 101 pure azimuth cells recorded when the animal was crawling on the horizontal arena, 40 pure pitch cells recorded on the vertical ring, and 5 conjunctive cells recorded both on the horizontal arena and on the vertical ring^19^. This ensured that that we could directly compare the tuning width and peak-firing rate in azimuth and pitch for pure and conjunctive populations.

### Choice of peak-firing rates and tuning widths

The experimentally recorded pure azimuth and pure pitch cells^19^ had similar peak-firing rates as computed from the above fit: $R_{{\mathrm{pure,azim}}}^{{\mathrm{data}}}$ = 0.98 ± 0.11 Hz, $R_{{\mathrm{pure,pitch}}}^{{\mathrm{data}}}$ = 1.17 ± 0.22 Hz (mean ± standard error of the mean, non-significant differences, *P* = 0.39 by Student’s *t*-test, Supplementary Fig. 10a). Because there was no significant difference in the firing rate of pure azimuth and pure pitch cells, the peak-firing rate was set to be the same for all pure neurons in the model, *R*_pure,model_ = 1.00 Hz (very close to the experimentally observed peak-firing rate averaged over all the recorded pure cells $R_{{\mathrm{pure}}}^{{\mathrm{data}}}$ = 1.04 ± 0.01 Hz).

The peak-firing rate of conjunctive cells (defined as the highest peak-firing rate of the two marginal tunings to azimuth and pitch) was significantly higher than that of all pure cells: $R_{{\mathrm{conj}}}^{{\mathrm{data}}}$ = 3.4 ± 1.6 Hz (*P* < 0.001 by Student’s *t*-test, Supplementary Fig. 10a).

Note that the peak-firing rates above are in fact the modulation depth of the tuning curves—i.e., the peak-firing rate minus the baseline firing rate—we report the modulation depth because some neurons had a non-zero baseline firing rate. However, in order to reduce the number of free parameters for the model we chose *c*_2_ = *c*_4_ = 0, corresponding to no baseline firing rate of pure and conjunctive cells.

Pure azimuth and pure pitch cells also had very similar tuning width, which is computed at half-height of the fitted tuning curve from baseline (differences are non-significant, *P* = 0.78 by Student’s *t*-test, Supplementary Fig. 10b). The tuning width of conjunctive cells was not significantly different from the tuning width of pure cells in the corresponding dimensions (*P* = 0.82 for azimuth, and *P* = 0.16 for pitch, by Student’s *t*-test), resulting in similar average tuning width in azimuth and pitch for both cell types (not significantly different by Student’s *t*-test, *P* = 0.48, Supplementary Fig. 10b). Therefore, for modeling purposes we treated the experimentally observed tuning width of pure and conjunctive cells to be the same. To strengthen the connection to other neuronal systems where tuning tends to be narrower than in the head-direction system, we chose a tuning of 45° (corresponding to *κ* = 9.11, equal for pure and conjunctive cells), unless noted otherwise. In Fig. 3, we analyzed a range of tuning widths to show that our results generalize across tuning widths, and included detailed results for the tuning width fitted to the head-direction data (*κ* = 2.37).

In the model, unless noted otherwise, the peak-firing rate of conjunctive cells was chosen such that each population (pure or conjunctive) emitted the same number of spikes on average over multiple presentations of the stimulus and multiple samples of the cells’ preferred directions (i.e., the mean population firing rates were the same). To achieve this, we computed the proportionality constant between *R*_conj,model_ and *R*_pure,model_ by integrating over the tuning curves of *N* cells. The proportionality constant depends on the tuning width parameter and the stimulus dimensionality *D*, and is given by *R*_conj,model_ = *R*_pure,model_ exp((*D* − 1)*κ*)/$I_0^{D - 1}(\kappa )$, where *I*_0_ is the modified Bessel function of the first kind. Similar integrals were performed in the calculation of the FI, and are shown below (Eqs. (5)–(10)).

We focused on this normalization condition because (1) we think that a comparison between two populations that emitted the same number of spikes is the most appropriate one to make, and (2) it matched almost precisely the experimental data, where we observed that the tuning width of both populations was very similar (Supplementary Fig. 10b), whereas the peak-firing rate of conjunctive cells was significantly elevated compared to the pure cells ($R_{{\mathrm{pure}}}^{{\mathrm{data}}}$ = 1.04 ± 0.01 Hz versus $R_{{\mathrm{conj}}}^{{\mathrm{data}}}$ = 3.4 ± 1.6 Hz, Supplementary Fig. 10a).

In addition to the peak-firing rate normalization that yields equal mean population firing rate for pure and conjunctive cells, we examined additional normalization conditions:

- One condition is the case where the peak-firing rate of conjunctive cells was chosen such that the two populations have equal FI. Under this condition the two populations have the same decoding error in the limit of large number of neurons and long decoding time, as explained below. To this end, we set the peak-firing rate of conjunctive cells to *R*_conj,model_ = *R*_pure,model_ exp((*D* − 1)*κ*)/$\left( {I_0^{D - 1}(\kappa )D} \right)$ (here again the firing depends on the tuning width and the dimensionality). This special case is discussed in Supplementary Fig. 3.
- To examine the effect of pure and conjunctive cells having different tuning width, we varied the tuning width of pure cells between 35 and 100°, while the tuning curve of conjunctive cells was fixed at 45°. The peak-firing rate of pure cells was chosen such that the two populations emitted the same number of spikes on average (Supplementary Fig. 6).

In the notation of Eqs. (1) and (2) these definitions correspond to setting *c*_1_ = $R_{{\mathrm{pure,model}}}{\kern 1pt} e^{ - \kappa _{{\mathrm{pure}}}}$ and *c*_3_ = $R_{{\mathrm{conj,model}}}{\kern 1pt} e^{ - 2\kappa _{{\mathrm{conj}}}}$.

### Choice of preferred direction and stimulus distributions

For simplicity, we chose for our simulations uniform distributions of preferred pitch and azimuth, for both pure and conjunctive cells. The empirical distributions found for both the preferred directions (i.e., the tuning) and for the sampled directions (i.e., the stimulus) are shown for this data set in Figs. 1d, f and 4c of Finkelstein et al.^19^ Importantly, in experiments where the entire range was sampled, tuning to both pitch and azimuth was uniform.

We also chose a uniform distribution of stimuli, although choosing any other stimulus distribution would not have changed our results given the uniform distribution of preferred angles. This is true because from the point of view of the decoder each stimulus is identical. In other words, the number of cells with preferred direction in the neighborhood of a given stimulus does not depend on the location of the stimulus, and therefore it also does not depend on the distribution from which the stimulus is drawn.

In the model, each neuron had Poisson statistics. Unless noted otherwise, there were no noise correlations, so the probability of observing *n*_1_,…, *n*_*N*_ spikes from neurons 1,…, *N* respectively during an interval of duration *T* given the stimulus (*φ*, *θ*) is:

3 $$p\left( {n_1, \ldots ,n_N|\varphi ,\theta } \right) = p\left( {n_1|\varphi ,\theta } \right) \times \ldots \times p\left( {n_N|\varphi ,\theta } \right) = \mathop {\prod}\limits_{i = 1}^N {\kern 1pt} p_i\left( {n_i|\varphi ,\theta } \right),$$

where,

4 $$p_i\left( {n_i|\varphi ,\theta } \right) = \frac{{\left[ {R_i(\varphi ,\theta )T} \right]^{n_i}}}{{n_i!}}e^{ - R_i(\varphi ,\theta )T}.$$

### Fisher information

We begin by computing the FI of a single population of neurons with 1D von-Mises tuning curves, and then extend it to conjunctive cells in two or more dimensions. The model used in Figs. 2–4 has *N*/2 pure azimuth and *N*/2 pure pitch neurons, and *N* conjunctive neurons, so we carry out the calculations for these population sizes.

The FI at a particular stimulus value *φ* is equal to a sum over all neurons of the squared derivative of the tuning curve, weighted by the inverse of the tuning curve:

5 $$J(\varphi ) = T\mathop {\sum}\limits_{i = 1}^{N/2} \frac{{\left( {\frac{{\mathrm{d}}}{{{\mathrm{d}}\varphi }}R_i(\varphi )} \right)^2}}{{R_i(\varphi )}}.$$

For large *N*, we can replace the sum over neurons (and their preferred head-directions) with an integral that includes the distribution of preferred directions. In this section, we assume a uniform distribution of preferred directions. Consequently, the FI is constant for all values of the stimulus *φ*, giving

6 $$J_{\varphi \varphi } = \frac{{(N{\mathrm{/}}2)R_{{\mathrm{pure}}}T}}{{2\pi }}{\int_0^{2\pi }} {\kern 1pt} {\mathrm{d}}\varphi {\int_0^{2\pi }} {\kern 1pt} {\mathrm{d}}\theta \frac{{\left( {\frac{{\mathrm{d}}}{{{\mathrm{d}}\varphi }}e^{\kappa ({\mathrm{cos}}\varphi - 1)}} \right)^2}}{{e^{\kappa ({\mathrm{cos}}\varphi - 1)}}},$$

where from now on we drop the model subscript on the parameters *R*_pure_, *R*_conj_. The notation *J*_*φφ*_ indicates that in the numerator of Eq. (6) the derivative of the tuning curve is taken twice with respect to *φ*, and hence that term is squared. Its usefulness will become apparent when we move on to treating the problem in dimensions larger than one. The integral over *θ* reminds us that the stimulus is two-dimensional, but it is equal to one because the cells are not tuned to this angle.

This integral can be carried out explicitly for von-Mises tuning curves, leading to

7 $$J_{{\mathrm{pure}}} = J_{\varphi \varphi } = \frac{1}{2}NR_{{\mathrm{pure}}}T\kappa e^{ - \kappa }I_1(\kappa )$$

where *I*_*ν*_(*κ*) denotes the *ν*-th order modified Bessel function of the first kind.

When the stimulus is multidimensional, the FI is no longer a scalar. Rather, it is a matrix in which the diagonal elements are similar to the one-dimensional case, where both derivatives are with respect to the same stimulus coordinate. The off-diagonal elements are terms where the two derivatives are with respect to different stimulus coordinates.

In the general multidimensional case, the Cramér–Rao bound is an inequality between the mean squared error and the eigenvalues of the inverse FI matrix. Throughout the paper, unless noted otherwise, we focus on the scalar error, equal to the square root of the sum of squared errors along each of the stimulus dimensions. We will see below that for the simplified model, which we focus on—with no noise correlations—the FI is proportional to the identity matrix (i.e., the off-diagonal elements are all zero, and the diagonal elements are all equal to each other) for both pure and conjunctive populations. This means that for this simplified model the CR bound is given in terms of a scalar FI quantity—which is the proportionality constant connecting the FI matrix to the identity matrix.

Since we assume that the tuning of pure and conjunctive cells is identical along each of the stimulus coordinates, the diagonal elements of the FI matrix are equal. For pure cells, this is simply equal to the quantity computed above in the 1D case. For two-dimensional conjunctive cells, the diagonal elements of the FI matrix are,

8 $$J_{\varphi \varphi } = J_{\theta \theta } = \frac{{NR_{{\mathrm{conj}}}T}}{{4\pi ^2}}{\int_0^{2\pi }} {\kern 1pt} {\mathrm{d}}\varphi {\int_0^{2\pi }} {\kern 1pt} {\mathrm{d}}\theta \frac{{\left( {\frac{{\mathrm{d}}}{{{\mathrm{d}}\varphi }}e^{\kappa ({\mathrm{cos}}{\kern 1pt} \varphi + {\mathrm{cos}}{\kern 1pt} \theta - 2)}} \right)^2}}{{e^{\kappa ({\mathrm{cos}}{\kern 1pt} \varphi + \cos {\kern 1pt} \theta - 2)}}}$$

leading to

9 $$J_{{\mathrm{conj}}} = J_{\varphi \varphi } = J_{\theta \theta } = NR_{{\mathrm{conj}}}T\kappa e^{ - 2\kappa }I_0(\kappa )I_1(\kappa ).$$

Under the assumption of no noise correlations, the off-diagonal elements of the FI matrix (i.e., the cross-terms *J*_*φθ*_, *J*_*θφ*_) are zero. Mathematically, one can see that taking a single derivative of the tuning curve with respect to each stimulus coordinate will result in an integrand that is an odd function of that coordinate, and hence that term will be zero.

Using the same approach one can also compute the average number of spikes emitted by each population, and find that

10 $$n_{{\mathrm{pure}}} = NTR_{{\mathrm{pure}}}{\kern 1pt} e^{ - \kappa }I_0(\kappa )\quad{\mathrm{and}}\quad n_{{\mathrm{conj}}} = NTR_{{\mathrm{conj}}}{\kern 1pt} e^{ - 2\kappa }I_0^2(\kappa ).$$

Throughout the paper, except in Supplementary Fig. 3, we normalize the peak-firing rate of conjunctive neurons *R*_conj_ such that the mean population firing rate of the pure and conjunctive populations is equal. We set *n*_pure_ = *n*_conj_, and using Eq. (10) this gives the following relationship between the peak-firing rates of pure and conjunctive cells (and the tuning width parameter *κ*):

11 $$R_{{\mathrm{conj}}} = R_{{\mathrm{pure}}}\frac{{e^\kappa }}{{I_0(\kappa )}}.$$

The Bessel function in the denominator arises from the integration over the von-Mises tuning curve. Conjunctive cells are tuned to both stimulus coordinates, thus one of the Bessel functions does not cancel out.

In the model on which we focus in this study, the FI matrix is proportional to the identity matrix for both pure and conjunctive populations. The proportionality constants (*J*_pure_, *J*_conj_) give the lower bound on the mean squared error for the decoding errors of these populations, and thus it is instructive to compare the (scalar) FI quantities when the population firing rate is equal for pure and conjunctive cells.

Substituting *R*_conj_ into Eq. (9) and comparing the FI of the pure and conjunctive populations we find,

12 $$J_{{\mathrm{conj}}} = 2J_{{\mathrm{pure}}}.$$

In Supplementary Fig. 3, we choose the peak-firing rate of conjunctive neurons *R*_conj_ by setting *J*_conj_ = *J*_pure_ (i.e., equal FI for pure and conjunctive cells). Using Eqs. (7), (9) this gives,

13 $$R_{{\mathrm{conj}}} = R_{{\mathrm{pure}}}\frac{{e^\kappa }}{{2I_0(\kappa )}},$$

so using Eq. (10), in this case the mean population firing rates satisfy

14 $$n_{{\mathrm{pure}}} = 2n_{{\mathrm{conj}}}.$$

Similar calculations can be carried out to compute the FI and the total firing rate of conjunctive neurons coding more than two dimensions (Fig. 3c, Supplementary Fig. 2). We normalized tuning curves such that the population firing rate of *N* conjunctive neurons coding a *D* dimensional variable is equal to *D* sub-populations of pure neurons, each of size *N*/*D*. When doing so we found that the FI ratio is

15 $$\frac{{J_{{\mathrm{conj}}}(D)}}{{J_{{\mathrm{pure}}}(D)}} = D.$$

### Decoder

Given the spike train *n*_1_,…, *n*_*N*_, the goal of the decoder is to find the head-direction (*φ*_est_, *θ*_est_) that most likely gave rise to the observed spike train. This is not a trivial exercise because of the stochastic nature of the spike trains. If the spike trains were deterministic, we would expect the decoder to recover the correct stimulus exactly.

One may wonder why the error of representing two or more different types of information jointly is relevant for an organism, and how to define the error in scenarios where the relevant variables are measured in different units (for example, the time and distance to the location of a future event). Animals must often act upon multiple streams of information by choosing a single strategy, so the accuracy with which all stimulus variables are represented jointly (i.e., the scalar error over all dimensions) can determine the success or failure of this strategy. In principle, the definition of the error itself can also follow from the fact that errors in the estimates of different stimulus components may lead to similar non-optimal outcomes. In other words, the stimulus components can be normalized along different dimensions such that equal deviations of each of the normalized coordinates lead to equal cost to the animal. Doing this in practice is of course a difficult problem, which is fortunately circumvented in the head-direction system in bats that we discuss here—where all the variables have the same units. The theoretical analysis in our study assumes that all stimulus variables are measured using the same units and that errors along each direction are equally important.

### Maximum likelihood

Given our assumption of Poisson statistics, one can show that the ML decoder solves the following minimization problem for two pure sub-populations:

16 $$\begin{array}{l}\varphi _{{\mathrm{est}}} = \mathop {{{\mathrm{argmin}}}}\limits_\varphi {\cal L}(\varphi ) = \mathop {{{\mathrm{argmin}}}}\limits_\varphi \mathop {\sum}\limits_{i = 1}^{N/2} \left[ {n_i\kappa {\kern 1pt} {\mathrm{cos}}\left( {\varphi - \varphi _i} \right) - TR_{{\mathrm{pure}}}{\kern 1pt} e^{\kappa [{\mathrm{cos}}(\varphi \,-\, \varphi _i) -\, 1]}} \right]\\ \theta _{{\mathrm{est}}} = \mathop {{{\mathrm{argmin}}}}\limits_\theta {\cal L}(\theta ) = \mathop {{{\mathrm{argmin}}}}\limits_\theta \mathop {\sum}\limits_{i = 1}^{N/2} \left[ {n_i\kappa {\kern 1pt} {\mathrm{cos}}\left( {\theta - \theta _i} \right) - TR_{{\mathrm{pure}}}{\kern 1pt} e^{\kappa [{\mathrm{cos}}(\theta \,-\, \theta _i) -\, 1]}} \right]\end{array}$$

and similarly for a conjunctive population:

17 $$\begin{array}{l}\left( {\varphi _{{\mathrm{est}}},\theta _{{\mathrm{est}}}} \right) = \mathop {{{\mathrm{argmin}}}}\limits_{\varphi ,\theta } {\cal L}(\varphi ,\theta )\\ {\cal L}(\varphi ,\theta ) = \mathop {\sum}\limits_{i = 1}^N \left[ {n_i\kappa \left[ {{\mathrm{cos}}\left( {\varphi - \varphi _i} \right) + {\mathrm{cos}}\left( {\theta - \theta _i} \right)} \right] - TR_{{\mathrm{conj}}}{\kern 1pt} e^{\kappa \left[ {{\mathrm{cos}}\left( {\varphi \,-\, \varphi _i} \right) \,+\, {\mathrm{cos}}\left( {\theta \,-\, \theta _i} \right) -\, 2} \right]}} \right].\end{array}$$

These equations were solved numerically using standard optimization algorithms, yielding ML estimated head-directions. The contour lines of the ratio of mean decoding errors using pure and conjunctive cells were used to define the regimes (in *N* − *T* space). The contour lines we plotted were at values $\sqrt D \pm \delta$ (except Supplementary Fig. 3 where we used 1 ± *δ*). The value of *δ* we used was 0.02 in all cases (Fig. 3a, b, Supplementary Figs. 2a, b and 7b). In Supplementary Figs. 4e, f and 5b, c, in the absence of an analytical derivation of the error ratio in the limit of large *N*, *T*, we used the value found at the largest *N*, *T* in the simulations as the boundary between the regimes.

From a mathematical point of view, the main problem we address in this study is the performance of this decoder when it uses the responses of pure cells or conjunctive cells. A key observation is that each of the functions ${\cal L}(\varphi ),{\cal L}(\theta ),{\cal L}(\varphi ,\theta )$ has two independent contributions. We can identify the first term as the so called population vector: a sum of the preferred angles scaled by the number of spikes each neuron fired (and possible constant factors). The variability of this term stems from the Poisson spiking statistics, i.e., from the fact that in each presentation of the stimulus, *n*_*i*_ is in general not equal to *T* times the average firing rate for the particular value of the stimulus. On the other hand, the second term is not affected by the stochasticity of spike generation. Rather, it corrects for the possibility that some stimulus values have more “nearby cells” (cells with preferred direction that is close to the stimulus) than other stimulus values. As the number of neurons *N* tends to infinity, all stimulus values are equally covered so that the second term becomes equal to the average population firing rate, and does not enter into the optimization procedure. Thus in this limit the ML estimate is equal to the population vector (PV) estimate.

### Population vector

The PV decoder is most readily understood geometrically. We can represent each neuron by a vector that points in its preferred head-direction, and is scaled by the number of spikes that neuron fired. The PV is the vector sum of all these “individual” vectors, and the estimated angle is the direction to which the PV is pointing.

### The periodic Cramér–Rao bound

In its classical formulation the CR bound deals with unbounded stimulus coordinates, such as position. In this case, the error too can grow unboundedly, as the FI goes to 0. Our manuscript focuses on decoding of angular variables which are bounded between 0 and 2*π*.

The difference is that as the FI goes to 0 (for fewer and fewer spikes) the CR bound states that the mean squared error should grow to infinity. This however cannot be the case, since the worst error a decoder can make is to “guess” the opposite direction, meaning that the mean squared error is bounded from above. This is the reason that the ratio of the decoding error and its lower bound according to the CR bound is non-monotonic (see Supplementary Fig. 1).

For a 2D stimulus as *T* decreases, the error of each pure cell subpopulation starts to saturate to its maximal value of 90° before the conjunctive cell error saturates to its maximal value of ~138° (the maximal average errors were computed assuming the decoded angle was completely random). To rigorously address this issue, Routtenberg & Tabrikian^58^ derived a so called periodic Cramér–Rao bound that takes into account the upper bound on the error—but their theory cannot be readily applied to our case since doing so requires knowing the distribution of errors made by a specific decoder.

### Noise correlations and non-independent models

We explored above the relative advantages of pure versus conjunctive coding of multidimensional stimuli assuming that cells’ spike counts are random variables that depend on the stimulus alone, and not on the response of other neurons. We now introduce dependencies between neurons, which are commonly referred to as noise correlations (NC). We consider four “strategies” of introducing dependencies between neurons’ responses: noise correlations, shared additive gain, shared multiplicative gain and shared inputs (“pooling”).

### Noise correlations

We assume a specific NC structure, i.e., the relationship between the overlap of two neurons’ tuning curves to the value of their pairwise noise correlations. For pure cells, we assume this structure is similar to that found in the head-direction system of rodents^25^. For conjunctive cells, we assume the same dependence, but use the distance between two-dimensional tuning curves instead of one-dimensional curves. We consider two possible decoders. First, we study the error using a “naive” decoder for which information about the dependencies between cells is not available to the downstream target. This decoder is therefore the same as the one used in the absence of NC. Second, we consider a decoder that infers the identity of the stimulus while taking into account the fact that the spike counts are correlated.

We emphasize that, in contrast to the work of Abbott & Dayan^23^ and a large literature on the subject of NC that followed, our focus here is not on whether the decoding accuracy is better or worse in the presence of NC (and possibly a specialized decoder). Rather, we are interested in asking whether NC affect the trade-off we found in the independent case between encoding by pure versus conjunctive cells.

For this set of assumptions, we found that the analysis of mixed-dimensionality coding remains largely unaffected when we introduce noise correlations. These results suggest that the benefits of a mixed-dimensionality code could potentially hold regardless of the presence or the structure of NC and the identity of the decoder (Supplementary Fig. 4).

The population coding model with both pure and conjunctive neurons that incorporates NC was constructed using the following procedure:

- Like the model with no NC, we drew the preferred directions of all cells independently from a uniform distribution.
- We computed the pairwise preferred direction distance matrix *d*_*ij*_ (Supplementary Fig. 4a),
  18 $$d_{ij} = \left\{ {\begin{array}{*{20}{l}} {\left| {\theta _i - \theta _j} \right|,} \hfill & {i,j{\kern 1pt} {\mathrm{are}}{\kern 1pt} {\mathrm{pure}}{\kern 1pt} {\mathrm{pitch}}} \hfill \\ {\left| {\varphi _i - \varphi _j} \right|,} \hfill & {i,j{\kern 1pt} {\mathrm{are}}{\kern 1pt} {\mathrm{pure}}{\kern 1pt} {\mathrm{azimuth}}} \hfill \\ {\sqrt {\left| {\theta _i - \theta _j} \right|^2 + \left| {\varphi _i - \varphi _j} \right|^2} ,} \hfill & {i,j{\kern 1pt} {\mathrm{are}}{\kern 1pt} {\mathrm{conjunctive}}} \hfill \end{array}} \right.$$
- From *d*_*ij*_, we computed the correlation matrix *c*_*ij*_, using a function similar to that found by Peyrache et al.^25^ These authors found that head-direction cells in rats are positively correlated if their preferred directions are similar (*d*_*ij*_ ≲ 45°), negatively correlated when *d*_*ij*_ ≈ 60°, and uncorrelated when *d*_*ij*_ > 60° (see Supplementary Fig. 4a). We assume that the same NC structure exists for conjunctive cells, giving:
  19 $$c_{ij} = \left\{ {\begin{array}{*{20}{l}} {\frac{1}{4}{\mathrm{cos}}\left( {2d_{ij}} \right)e^{ - d_{ij}^2}} \hfill & {i \,\ne\, j} \hfill \\ 1 \hfill & {i = j} \hfill \end{array}} \right.$$
  We also assume that pure azimuth, pure pitch and conjunctive cells are uncorrelated with cells in other sub-populations.
- The final step is to draw spike counts with the specified correlation matrix. Since there is no closed form prescription for drawing a set of Poisson random numbers with an arbitrary correlation matrix^59^, we use a Gaussian approximation:
  - Draw a set of Gaussian random numbers *z*_*i*_ with mean 0 and correlation given by the matrix with elements *c*_*ij*_.
  - Compute the cumulative Gaussian distribution function of the *z*_*i*_’s, denoted *q*_*i*_.
  - The spike count of the neuron *i*, denoted *n*_*i*_ is then the inverse cumulative Poisson distribution function of *q*_*i*_.

We verified numerically that the Gaussian approximation does not strongly distort the correlation structure, such that the resulting spike counts have Poisson statistics (guaranteed by using the inverse Poisson cumulative distribution function) and that their correlation matrix is approximately equal to *c*.

The simulation is completed by inferring the stimulus using the spike counts. We did that first by using a “naive” decoder that does not take into account the noise correlations (see Eqs. (16, 17)). Second, we used a decoder that infers the stimulus based on the spike counts and knowledge of the NC. Here, one cannot write an explicit expression for the likelihood of a set of *N* spike counts given the stimulus. This is for the same reason that one cannot directly draw Poisson distributed spike counts with a specific correlation structure. Briefly, for a Poisson distribution, the probability of a vector of spike counts depends on the covariance matrix, which itself depends on the spike counts, making it impossible to obtain an explicit form for the distribution. The same is not true for a Gaussian distribution in which the covariance is independent of the spike counts. We thus used the derivations and the expressions which appear in Ecker et al.^60^ for the Gaussian approximation of the likelihood function.

In Supplementary Fig. 4c, we show that the same qualitative behavior we observed in simulations of populations without noise correlations are also observed when comparing the performance of pure and conjunctive cells that do have NC as specified above. The value to which the error ratio saturates at large *N* and *T* is no longer $\sqrt 2$ because the Fisher information of each population depends on the NC, so its ratio changes too. For the naive decoder, this ratio depends on *N* in a non-trivial way—it saturates slowly with *N* relative to the saturation of the error ratio for the NC dependent decoder (compare top and bottom panels of Supplementary Fig. 4d).

### Shared additive gain

We consider here the possibility that on a given “trial,” a subpopulation of pure cells, or the population of conjunctive cells can be upregulated or downregulated in a shared manner. To this end, we repeated the simulations with the following modification.

On each trial, the tuning curve of all pure azimuth, pure pitch and conjunctive cells was shifted by random amounts Δ_pure,azimuth_, Δ_pure,pitch_ and Δ_conj_, respectively. The Δ’s were drawn independently from a uniform distribution between −0.2 and 0.2, such that the shifted tuning curves for pure azimuth, pure pitch, and conjunctive cells are (compare to Eqs. (1), (2)):

20 $$\begin{array}{l}R_i(\varphi ) = R_{{\mathrm{pure}}}{\kern 1pt} \left( e^{\kappa \left[ {{\mathrm{cos}}(\varphi - \varphi _i) - 1} \right]} + {\mathrm{\Delta }}_{{\mathrm{pure,azimuth}}} \right)\\ R_i(\theta ) = R_{{\mathrm{pure}}}{\kern 1pt}\left( e^{\kappa \left[ {{\mathrm{cos}}(\theta - \theta _i) - 1} \right]} + {\mathrm{\Delta }}_{{\mathrm{pure,pitch}}}\right)\\ R_i(\varphi ,\theta ) = R_{{\mathrm{conj}}}{\kern 1pt} \left( e^{\kappa \left[ {{\mathrm{cos}}(\varphi - \varphi _i) + {\mathrm{cos}}(\theta - \theta _i) - 2} \right]} + {\Delta }_{{\mathrm{conj}}} \right)\end{array}$$

Spikes were then drawn from a Poisson distribution with parameter equal to *T* times the shifted tuning curve evaluated at the stimulus presented at that given trial. When that parameter was less than 0 (in cases where a population was downregulated) the parameter was set to 0.

Decoding was performed using the same ML decoder used in the simulations without shared variability. This corresponds to a situation where downstream targets do not have access to the value of the shared gain on a given trial.

### Shared multiplicative gain

We similarly considered a case where the shared gain was multiplicative instead of additive. In this case, each tuning curve was multiplied by a random number (shared among neurons in the same subpopulation) *α*_pure,azimuth_, *α*_pure,pitch_ and *α*_conj_. The *α*’s were drawn independently from a log-normal distribution with parameters *μ* = −1/2, *σ*^2^ = 1, such that the average multiplicative gain factor was 1. Now the tuning curves are,

21 $$\begin{array}{l}R_i(\varphi ) = \alpha _{{\mathrm{pure,azimuth}}}R_{{\mathrm{pure}}}e^{\kappa \left[ {{\mathrm{cos}}(\varphi \,-\, \varphi _i) - 1} \right]}\\ R_i(\theta ) = \alpha _{{\mathrm{pure,pitch}}}R_{{\mathrm{pure}}}e^{\kappa \left[ {{\mathrm{cos}}(\theta \,-\, \theta _i) - 1} \right]}\\ R_i(\varphi ,\theta ) = \alpha _{{\mathrm{conj}}}R_{{\mathrm{conj}}}e^{\kappa \left[ {{\mathrm{cos}}(\varphi \,-\, \varphi _i) + cos(\theta \,-\, \theta _i) -\, 2} \right]}\end{array}$$

Again, spikes were then drawn from a Poisson distribution with parameter equal to *T* times the tuning curves evaluated at the stimulus presented at that given trial; and decoding was performed using the a ML decoder that is unchanged by the gain modulation.

### Shared input (feed-forward pooling model)

To test whether the same trade-off between coding by pure/conjunctive cells exists when the conjunctive representation is generated in a more realistic fashion (relative to pre-defined tuning curves and NC structure) we constructed the following pooling model.

Two populations of size *N*_0_ have von-Mises tuning curves (*κ* = 9.1, corresponding to tuning width of 45°) and evenly spaced preferred orientations *θ*_*j*_ = *φ*_*j*_ = ${\textstyle{{2\pi j} \over N}}$, *j* = 1,…, *N*. Spike counts (denoted $m_j^\theta$, $m_j^\varphi$) are produced independently with Poisson statistics using integration time *T* (which will be varied in the same way as was done for the rest of the models). For the tuning preferences of the downstream populations, in every simulation, we chose uniformly at random *N*/2 azimuth directions $\left( {\theta _i^{{\mathrm{pure}}}} \right)$, *N*/2 pitch directions $\left( {\varphi _i^{{\mathrm{pure}}}} \right)$ and *N* pairs of azimuth-pitch directions ($\theta _i^{{\mathrm{conj}}}$, $\varphi _i^{{\mathrm{conj}}}$).

Given the spike counts of the upstream populations (the first layer, see schematic in Supplementary Fig. 5) and the preferred orientations of the downstream populations, we computed the spike rates of neurons in the downstream populations (i.e., the second layer) using the following equations, which describe the pooling operation that pure azimuth, pure pitch and conjunctive neurons perform on their shared inputs:

22 $$\begin{array}{l}r_{i,\varphi }^{{\mathrm{pure}}} = A^{{\mathrm{pure}}}\mathop {\sum}\limits_{j = 1}^{N_0} {\kern 1pt} m_j^\varphi {\kern 1pt} {\mathrm{exp}}\left[ {\kappa {\kern 1pt} {\mathrm{cos}}\left( {\varphi _i^{{\mathrm{pure}}} - \frac{{2\pi j}}{N}} \right)} \right],\\ r_{i,\theta }^{{\mathrm{pure}}} = A^{{\mathrm{pure}}}\mathop {\sum}\limits_{j = 1}^{N_0} {\kern 1pt} m_j^\theta {\kern 1pt} {\mathrm{exp}}\left[ {\kappa {\kern 1pt} {\mathrm{cos}}\left( {\theta _i^{{\mathrm{pure}}} - \frac{{2\pi j}}{N}} \right)} \right],\\ r_i^{{\mathrm{conj}}} = \frac{{A^{{\mathrm{conj}}}}}{T}\left( {\mathop {\sum}\limits_{j = 1}^{N_0} {\kern 1pt} m_j^\theta {\kern 1pt} {\mathrm{exp}}\left[ {\kappa {\kern 1pt} {\mathrm{cos}}\left( {\theta _i^{{\mathrm{conj}}} - \frac{{2\pi j}}{N}} \right)} \right]} \right)\left( {\mathop {\sum}\limits_{j = 1}^{N_0} {\kern 1pt} m_j^\varphi {\kern 1pt} {\mathrm{exp}}\left[ {\kappa {\kern 1pt} {\mathrm{cos}}\left( {\varphi _i^{{\mathrm{conj}}} - \frac{{2\pi j}}{N}} \right)} \right]} \right).\end{array}$$

The constants *A*^pure^, *A*^conj^ were chosen such that the population firing rate of the pure downstream population and the conjunctive downstream populations were equal. Since both $m_j^\theta$, $m_j^\varphi$ are proportional to *T*, dividing by *T* in the definition of $r_i^{{\mathrm{conj}}}$ ensures that the expected number of spikes of conjunctive neurons downstream is also linear in *T* (instead of quadratic if this factor was not included). The spike counts of neurons in the main (downstream) populations are drawn independently from a Poisson distribution,

23 $$n_{i,\theta }^{{\mathrm{pure}}}\sim {\mathrm{Poisson}}\left( {r_{i,\theta }^{{\mathrm{pure}}}} \right),\,n_{i,\varphi }^{{\mathrm{pure}}}\sim {\mathrm{Poisson}}\left( {r_{i,\varphi }^{{\mathrm{pure}}}} \right),\,n_i^{{\mathrm{conj}}}\sim {\mathrm{Poisson}}\left( {r_i^{{\mathrm{conj}}}} \right).$$

Since the responses of the main population are produced in two stages, there is no explicit form for the likelihood functions $P\left( {\left\{ {n_{i,\varphi }^{{\mathrm{pure}}}} \right\}_{i = 1, \ldots ,N/2}|\varphi } \right)$, $P\left( {\left\{ {n_{i,\theta }^{{\mathrm{pure}}}} \right\}_{i = 1, \ldots ,N{\mathrm{/}}2}|\theta } \right)$, $P\left( {\left\{ {n_i^{{\mathrm{conj}}}} \right\}_{i = 1, \ldots ,N}|\phi ,\theta } \right)$ making it unfeasible to use a ML decoder. We thus decoded the stimulus using the population vector. We set the error to *π* when there were no spikes for a given population.

A schematic illustrating the model is shown in Supplementary Fig. 5a. Despite the fact that the Poisson noise in both stages (upstream and main) is independent, pooling from shared inputs introduces correlations between the spike counts of neurons in the second layer in a biologically plausible way. In this model, correlations between neurons both within each population as well as across populations. The magnitude of the noise correlations in this model depends on the relative size of the upstream and downstream populations (*N*_0_ and *N*, respectively): when *N* is close to *N*_0_ correlations become larger.

We computed the decoding error ratio for the main pure and conjunctive populations. Plotting the error ratio in the *N* − *T* space, we clearly find two regimes where the ratio is bigger than or smaller than the ratio for large *N*, *T*, which is ~0.8 for the parameters we use (Supplementary Fig. 5a, b). This means that if conjunctive cells pool broadly from an upstream population of pure cells, they can outperform a population of pure cells of the same size and tuning properties. This finding does not depend on the magnitude of noise correlations created by pooling, since the same behavior of the error ratio is seen for *N*_0_ = 10,000 (Supplementary Fig. 5b, large upstream population compared to the maximal size of the downstream population *N* = 2000) and for *N*_0_ = 4000 (Supplementary Fig. 5c, upstream population of comparable size to the maximal size of the downstream population).

### Expected error of a mixed pure and conjunctive population

In Supplementary Fig. 8, we show the error of a mixed population of pure and conjunctive cells as a function of the fraction of cells that have pure tuning. This error is minimal when all the cells are conjunctive (Supplementary Fig. 8a).

However, we argue in the text that the error of the mixed population $\epsilon _{{\mathrm{mix}}}$ should be compared to $\epsilon _{{\mathrm{mix,independent}}}$, the error of a mixed population assuming the pure and conjunctive sub-populations contribute independently to reducing the error, but have no “synergistic” interactions. Here we derive $\epsilon _{{\mathrm{mix,independent}}}$ as a function of the errors $\epsilon _{{\mathrm{pure}}}$ and $\epsilon _{{\mathrm{conj}}}$ of the pure and conjunctive sub-populations.

We assume that the inverse of the squared errors are information-like quantities, in the sense that they are additive: The FI provided about a stimulus by two sub-populations jointly is equal to the sum of the FI provided about the stimulus by the two sub-populations separately. When the error is not saturated to the CR bound, the appropriate quantity is the inverse of the squared error, which is smaller than the actual FI (effectively, less information about the stimulus exists in the spiking response).

Mathematically,

24 $$\tilde J_{{\mathrm{pure}}} = \frac{1}{{\epsilon _{{\mathrm{pure}}}^{\mathrm{2}}}},\,\tilde J_{{\mathrm{conj}}} = \frac{1}{{\epsilon _{{\mathrm{conj}}}^{\mathrm{2}}}}.$$

Note that $\tilde J_{{\mathrm{pure}}}$ and $\tilde J_{{\mathrm{conj}}}$ are not the Fisher information, because we do not assume that $\epsilon _{{\mathrm{pure}}}$ and $\epsilon _{{\mathrm{conj}}}$ are saturated to the CR bound.

The total information about the stimulus provided by these two sub-populations is equal to $\tilde J_{{\mathrm{mix,independent}}}$ = $\tilde J_{{\mathrm{pure}}} + \tilde J_{{\mathrm{conj}}}$, which corresponds to the inverse of the expected squared error from the mixed population, giving

25 $$\epsilon _{{\mathrm{mix,independent}}} = \frac{1}{{\sqrt {\tilde J_{{\mathrm{mix,independent}}}} }} = \frac{{\epsilon _{{\mathrm{pure}}}\epsilon _{{\mathrm{conj}}}}}{{\sqrt {\epsilon _{{\mathrm{pure}}}^{\mathrm{2}} + \epsilon _{{\mathrm{conj}}}^{\mathrm{2}}} }}.$$

### Multidimensional error and errors along each dimension

In Fig. 4a of the main text we show that for pure and conjunctive populations, the ratio of the total decoding error in 2D and the error along each of the directions independently approaches a value of 1.57 for large *T*. To show this, assume that the error is a Gaussian random variable with mean zero (since the decoder is unbiased) and variance *σ*. In general *σ* is different for pure and conjunctive cells and depends on *N* and *T*. The probability distribution of error $\epsilon _{}^{}$ along one either dimension is

26 $$p(\epsilon ) = p\left( {\epsilon _\varphi } \right) = p\left( {\epsilon _\theta } \right) = \frac{1}{{\sqrt {2\pi \sigma ^2} }}{\mathrm{exp}}\left( { - \frac{{\epsilon ^2}}{{2\sigma ^2}}} \right)$$

The distribution of the 2*D* error is

27 $$p\left( {\epsilon _\varphi ,\epsilon _\theta } \right) = \frac{1}{{2\pi \sigma ^2}}{\mathrm{exp}}\left( { - \frac{{\epsilon _\varphi ^2 + \epsilon _\theta ^2}}{{2\sigma ^2}}} \right).$$

We can then use this to compute the total mean error

28 $$\left\langle {\epsilon _2} \right\rangle = \frac{1}{{2\pi \sigma ^2}}\int\!\!\int_{ - \infty }^{ \infty } {\kern 1pt} {\mathrm{d}}\epsilon _\varphi {\mathrm{d}}\epsilon _\theta {\kern 1pt} {\mathrm{exp}}\left( { - \frac{{\epsilon _\varphi ^2 + \epsilon _\theta ^2}}{{2\sigma ^2}}} \right)\sqrt {\epsilon _\varphi ^2 + \epsilon _\theta ^2} = \sqrt {\frac{\pi }{2}} \sigma ,$$

and the error in one dimension

29 $$\left\langle {\epsilon}_{1} \right\rangle = \frac{1}{\sqrt {2\pi \sigma^{2}}}\int_{ - \infty }^{\infty}{\kern 1pt} {\mathrm{d}}\epsilon {\kern 1pt} {\mathrm{exp}}\left( { - \frac{\epsilon }{2\sigma^{2}}} \right)\left| \epsilon \right| = \sqrt {\frac{2}{\pi }} \sigma.$$

So the ratio is

30 $$\frac{{\left\langle {\epsilon _2} \right\rangle }}{{\left\langle {\epsilon _1} \right\rangle }} = \frac{\pi }{2} \approx 1.57,$$

independent of *σ*.

In Fig. 4a of the main we text show that this ratio is larger than *π*/2 for pure cells at short decoding times *T*. This results from the high probability of very large errors along at least one direction (azimuth or pitch), compared to the probability one would expect if the distribution of errors was Gaussian. Specifically, this is a signature of an error distribution with large kurtosis.

### Conditional error calculations at short decoding time

We have shown that for small *T* there is a regime where the conjunctive cells outperform the pure cells compared to what is expected from the FI considerations (i.e., in 2D, $\epsilon _{{\mathrm{pure}}}{\mathrm{/}}\epsilon _{{\mathrm{conj}}} > \sqrt 2$). In Supplementary Note 1 we discuss in detail the departure of the error from the Cramér–Rao bound. Here we explain the existence of this regime by computing the decoding errors conditioned on the number of spikes.

When the number of spikes emitted by the entire population is small (≲15), there is a finite chance that one of the pure sub-populations will fire very few spikes (≲5) such that estimation head-direction along one direction is very poor. In these instances, a decoder relying on the conjunctive population (that emits the same number of spikes on average) is likely to outperform one that relies on the pure cells.

To test whether the instances of poor estimates in one pure subpopulation explain this effect, we computed the probability that one of the pure sub-populations and the conjunctive population will fire a small number of spikes denoted by *n*_min_ (Supplementary Fig. 7a), and the corresponding conditional average errors:

$$\begin{array}{*{20}{l}} {\epsilon _{{\mathrm{conj}}}(n)} \hfill & {{\mathrm{decoding}}{\kern 1pt} {\mathrm{error}}{\kern 1pt} {\mathrm{from}}{\kern 1pt} {\mathrm{the}}{\kern 1pt} {\mathrm{conjunctive}}{\kern 1pt} {\mathrm{population}}} \hfill \\ {} \hfill & {{\mathrm{given}}{\kern 1pt} {\mathrm{that}}{\kern 1pt} {\mathrm{it}}{\kern 1pt} {\mathrm{has}}{\kern 1pt} {\mathrm{emitted}}{\kern 1pt} {\mathrm{at}}{\kern 1pt} {\mathrm{least}}{\kern 1pt} {\mathrm{2}}n_{{\mathrm{min}}}{\kern 1pt} {\mathrm{spikes}}} \hfill \\ {\epsilon _{{\mathrm{pure}}}(n)} \hfill & {{\mathrm{decoding}}{\kern 1pt} {\mathrm{error}}{\kern 1pt} {\mathrm{from}}{\kern 1pt} {\mathrm{the}}{\kern 1pt} {\mathrm{pure}}{\kern 1pt} {\mathrm{population}}{\kern 1pt} {\mathrm{given}}} \hfill \\ {} \hfill & {{\mathrm{that}}{\kern 1pt} {\mathrm{each}}{\kern 1pt} {\mathrm{subpopulation}}{\kern 1pt} {\mathrm{has}}{\kern 1pt} {\mathrm{emitted}}{\kern 1pt} {\mathrm{at}}{\kern 1pt} {\mathrm{least}}{\kern 1pt} n_{{\mathrm{min}}}{\kern 1pt} {\mathrm{spikes}}} \hfill \end{array}$$

For the comparison between the decoding errors from the pure and conjunctive populations to remain “fair,” the average firing rate should stay the same after instances with few spikes are removed. That is the reason that instances when the conjunctive population emitted <2*n*_min_ spikes were ignored.

The case *n*_min_ = 0 includes all instances because each population fired at least zero spikes in repeated simulation. As *n* is increased, additional “problematic” instances are ignored, and regime #3 disappears. For *n*_min_ = 4, the effect is completely eliminated (any remaining blue region for *n*_min_ = 3 in Supplementary Fig. 7b is within the error bars). In other words, regime #3 (where decoding from conjunctive cells leads to a smaller error than decoding from pure cells) is eliminated if instances where either of the pure sub-populations emitted less than three spikes are not taken into account when computing the average error.

Thus we conclude that the improved accuracy of a decoder that uses the conjunctive cells stems from the finite chance that when the number of spikes is small, their distribution among the two pure sub-populations will be uneven, leading to a very poor estimate.

### Flight kinematics during natural orientation behavior

We analyzed the behavioral data collected by Tsoar et al.^26^, which included a GPS tracking of 45 Egyptian fruit bats (*Rousettus aegyptiacus*) using lightweight GPS dataloggers. The GPS tracking data included all the bats that were reported in Tsoar et al.^26^, as well as several additional animals. Figure 5a, and Supplementary Movie 1 were made from this data set using Google Earth Software (Google Earth Pro, desktop version: https://www.google.com/earth/desktop/). This data set allowed us to compute the 3D coordinates of the bat’s position in space (*x*, *y*, *z*) at a 1 Hz sampling rate. We first computed the direction of the bat’s heading in 3D space from the positional estimate *x*, *y*, *z*. Specifically, we computed the heading-direction in azimuth (*φ*) and pitch (*θ*) of freely flying bats using the following equations:

31 $$\varphi = {\mathrm{angle}}\left( {{\mathrm{\Delta }}x + i{\mathrm{\Delta }}y} \right)$$

32 $$\theta = {\mathrm{angle}}\left( {\sqrt {{\mathrm{\Delta }}x^2 + {\mathrm{\Delta }}y^2} + i{\mathrm{\Delta }}z} \right)$$

where Δ*x*, Δ*y*, Δ*z* are the changes in the animal’s position between consecutive video frames; and *i* is the imaginary unit. The angular velocities in azimuth (*v*_*θ*_) and pitch (*v*_*φ*_) were computed by taking the absolute values of the first derivative of the azimuth and pitch heading-direction, respectively. The combined angular velocity in azimuth and pitch was defined as:

33 $$v_{\theta \times \varphi } = \sqrt {v_\theta ^2 + v_\varphi ^2} .$$

We included only data-points in which the animal was flying at instantaneous speeds larger than 0.5 m s^−1^ (to exclude epochs in which the animal hung stationary on the fruit-tree). Horizontal displacement was defined as the Euclidean distance traversed by the bat in the horizontal (*x*, *y*) plane in a time interval of 20s.

### Head-direction tuning at different angular velocities

We analyzed the behavioral and the electrophysiological data previously reported in Finkelstein et al.^19^ A total of 266 well-isolated neurons were recorded from dorsal presubiculum of 4 crawling bats. Head-direction in azimuth and pitch in crawling bats was computed using video tracking as described in Finkelstein et al.^19^ The angular velocities in azimuth (*v*_*θ*_) and pitch (*v*_*φ*_) were computed by taking the absolute difference in the head-direction angle (azimuth or pitch, respectively) between consecutive video frames, and multiplying it by the frame rate. The combined angular velocity in azimuth and pitch during crawling was computed using Eq. (33) above.

For the analysis presented in Fig. 6, we separated the behavioral data during crawling into two bins of low or high combined angular velocity, using a cutoff threshold of 10° s^−1^ (Fig. 6b). We chose this cutoff value because it could distinguish between navigational and maneuvering modes observed during natural behavior in the wild (Fig. 5b). We included for analysis a total of 127 cells, based on the following criteria: (i) Each of the two angular velocity bins (slow bin [<10° s^−1^] or fast bin [≥10° s^−1^]), included a minimum of 120 s of data; (ii) In each of these angular-velocity bins, the cell emitted at least 50 spikes. For cells that were recorded for two behavioral sessions, we chose the session with the largest number of spikes for all further analyses (single session per neuron).

For the analysis presented in Supplementary Fig. 9, we used adaptive binning in angular velocity. Specifically, we binned the behavioral distribution of angular velocities (Fig. 6b) into 4 bins with equal occupancy (25% of the behavioral data in each bin). Because each bin contained only a fraction of the data, we included only cells with high spike count for this analysis (more than 400 spikes per session, *n* = 134 cells out of the original 266 cells). For cells that were recorded for two behavioral sessions, we chose the session with the largest number of spikes for all further analyses (i.e., single session per neuron). In Supplementary Fig. 9a, e, we used 4 non-overlapping bins (each containing 25% of the behavioral data). In Supplementary Fig. 9b–d, f, we analyzed the data continuously as a function of angular velocity, using adaptive binning, where each bin also contained 25% of the behavioral data (as in Supplementary Fig. 9a), but the bin was moved in small increment steps of 1° s^−1^.

Head direction tuning in azimuth, pitch, or azimuth × pitch was computed as described in Finkelstein et al.^19^, with the key difference being that here we computed the tuning separately for different angular-velocity bins. Briefly, we first constructed 1D tuning curves separately for azimuth and pitch tuning, by dividing the number of spikes emitted by the cell in each angular bin by the time-spent in that bin during the relevant behavioral epoch (low or high angular-velocity). The directional tuning of the azimuth tuning was quantified by computing the Rayleigh vector length of the circular distribution. To quantify the directional tuning to pitch, for which we did not have a fully circular behavioral sampling, we computed the tuning width in pitch. Tuning width for both azimuth and pitch was defined as the tuning width at half-height, after subtracting the baseline firing rate^19^. To determine whether a cell had a significant directional tuning in each angular-velocity bin in any of the dimensions, we used shuffling analysis on the 1D tuning curves that were computed separately for the azimuth and pitch dimensions^19^. Neurons with significant tuning to only one of the dimensions (i.e., azimuth-only or pitch-only) were classified as pure cells, whereas neurons with significant tuning to both azimuth and pitch dimensions were classified as conjunctive cells. Note that a neuron could have a different classification in slow or fast angular-velocity bins (Fig. 6, Supplementary Fig. 9). In the analysis presented in Supplementary Fig. 9, tuning curves were computed using on average 25% of the behavioral data in each angular-velocity bin. To avoid spurious tuning due to sub-sampling, we defined a tuning to be significant in a particular angular-velocity bin if: (i) it passed the significance criterion according to the shuffling analysis described above—for this particular angular-velocity bin; and (ii) was stable for the duration of the session. A cell was considered to have a stable tuning in a particular angular-velocity bin if the tuning curves constructed separately for odd or even seconds of the data in this bin had a Pearson correlation coefficient of *r* > 0.25.

### Code availability

We used MATLAB code for computer simulation. The code will be made available on request.

## Electronic supplementary material

Supplementary Information

Description of Additional Supplementary Files

Supplementary Movie 1

## Data Availability

The data are archived on the Weizmann Institute of Science servers and will be made available on request.
