# Supplementary material for: Optimal dynamic coding by mixed-dimensionality neurons in the head-direction system of bats
Source: Nat Commun. 2018 Sep 4;9:3590. doi: 10.1038/s41467-018-05562-1 (PMC6123463; doi:10.1038/s41467-018-05562-1)
Supplement: Supplementary file 1 — Supplementary Information [file 41467_2018_5562_MOESM1_ESM.pdf]

Supplementary Information for

Optimal dynamic coding by mixed-dimensionality neurons in the head-direction system of bats

Finkelstein, Ulanovsky, Tsodyks, Aljadeff (2018) *Nature Communications*

Contents

	Page
**Supplementary Figures**	**2**
**Supplementary Note 1**	**16**
**Supplementary References**	**22**

Supplementary Figures

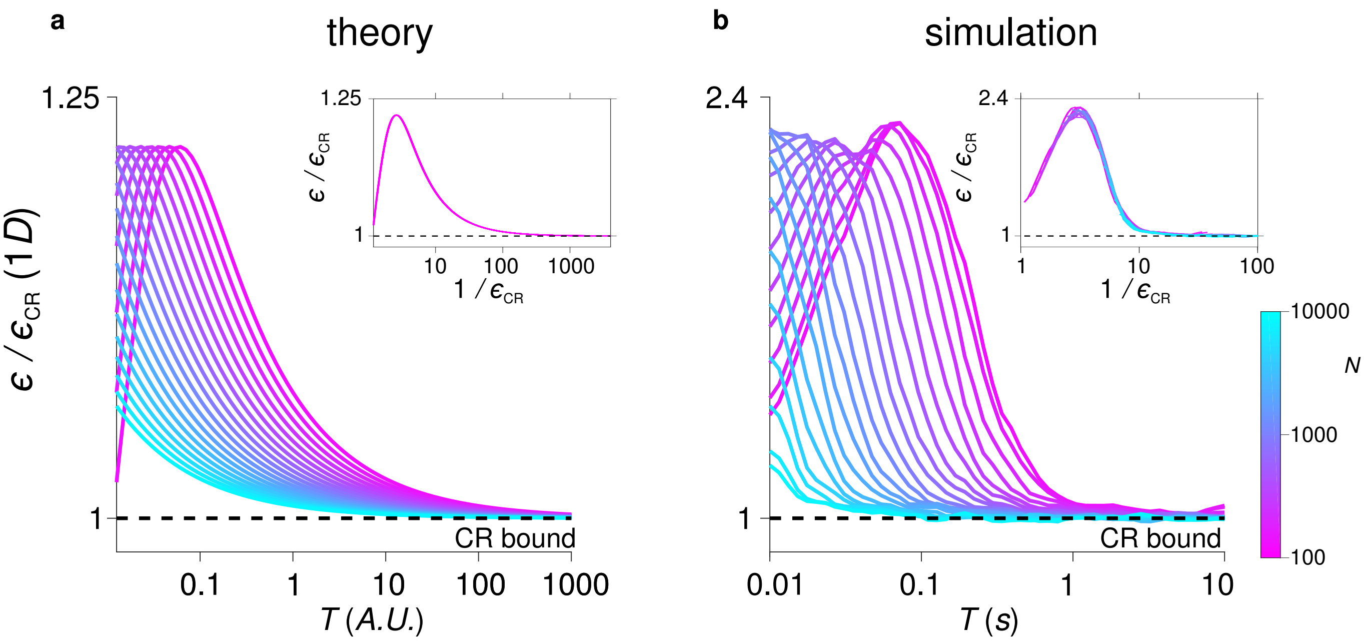

Supplementary Figure 1. Decoding accuracy of a 1D stimulus for finite N and T: saturation rate to the Cramér-Rao bound.

(**a**,**b**) For a 1D stimulus we compared here our theoretical derivation of the decoding error ϵ normalized by $\epsilon_{\rm CR}$ (the Cramér-Rao bound) (**a**, see Supplementary Note 1 for details) to the results of numerical simulations of a Maximum Likelihood decoder (**b**). Each line indicates a different population size N ranging between 100 and 10000 with logarithmic jumps (see color-bar). The theory correctly predicts that for short decoding time T the decoding error does not saturate the Cramér-Rao bound, and that it further diverges from this bound as N gets smaller (magenta-colored lines). The non-monotonic behavior of $\epsilon/\epsilon_{\rm CR}$ results from the fact that the average estimation error of an angular variable cannot exceed 90°, while $\epsilon_{\rm CR}$ which is computed from the FI does exceed 90° for small N and T (Supplementary Note 1). Insets: plotting the relative errors $\epsilon/\epsilon_{\rm CR}$ as function of $1/\epsilon_{\rm CR} = \sqrt{\rm FI}$ collapses the curves corresponding to different values of N onto one line, in agreement with our analysis. Since $1/\epsilon_{\rm CR} = \sqrt{\rm FI}$ is proportional to $\sqrt{N \times T}$, this means that in 1D, for large N, the error depends only on the product $N \times T$ and not on N and T separately.

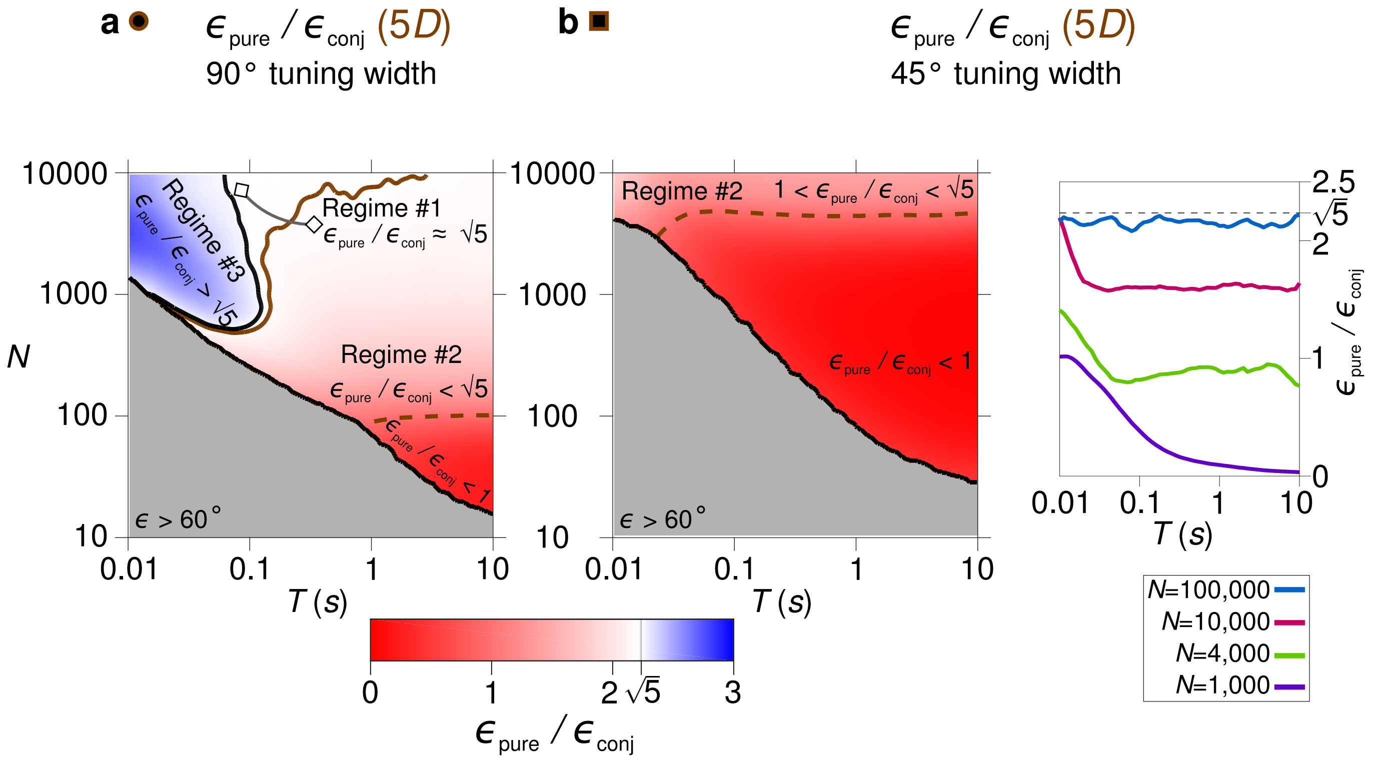

Supplementary Figure 2. Relative coding accuracy of pure and conjunctive cells representing a 5D variable.

We plot the error ratio of pure and conjunctive cells decoding a hypothetical 5D stimulus. Similar to the 2D case (**Fig. 3**), we identified here three regimes where the error ratio is equal to, smaller than and larger than the ratio predicted by the FI for large N and T – which in this case equals $\epsilon_{\text{pure}}/\epsilon_{\text{conj}} = \sqrt{5}$. (**a**) For wide tuning (90°), the range of N and T values we used is sufficient to find these regimes and the error ratio in $N - T$ space looks very similar to that in 2D (**Fig. 3**). (**b**) For narrow tuning (45°) conjunctive cells suffer from very poor coverage of this high-dimensional stimulus space. Thus, for the range of N and T values we used, we can only see regime #1 where $\epsilon_{\text{pure}}/\epsilon_{\text{conj}} \approx \sqrt{5}$ (blue line corresponding to $N = 10^5$, right panel) and regime #2 where $\epsilon_{\text{pure}}/\epsilon_{\text{conj}} < \sqrt{5}$ (left panel). The panel on the right shows the ratio of the decoding error by pure versus conjunctive cells, as a function of T, for 4 different population sizes (N). Note that for a 5D stimulus, pure cells can outperform conjunctive cells even at large N, and the conjunctive cells will not be accurate unless the population size is very large (above 5,000 neurons) or the decoding time is very short (the green line in the inset [$N = 4,000$] is mostly below 1) . The color bar indicates the error-ratio between pure and conjunctive cells for a 5D stimulus.

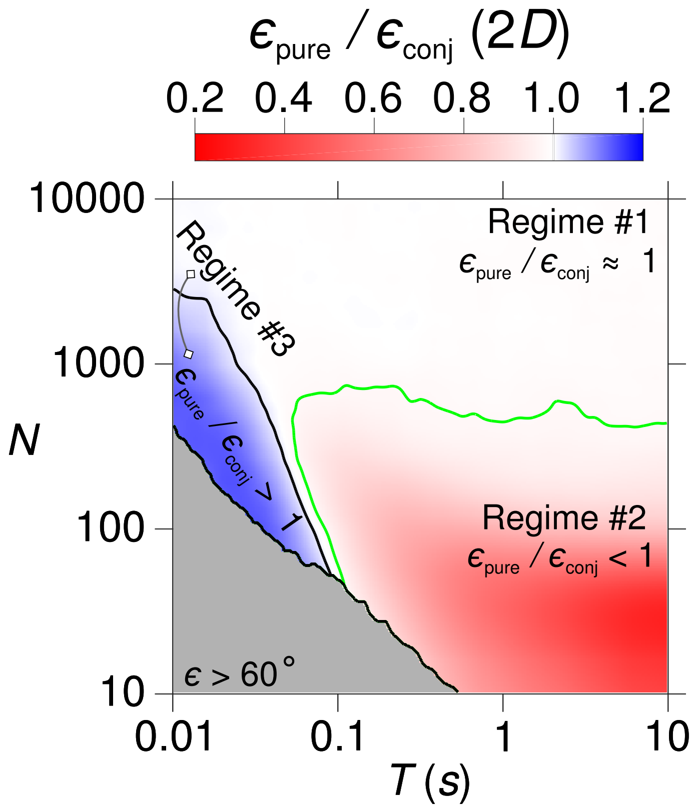

Supplementary Figure 3. Relative decoding performance of pure versus conjunctive cells for different scaling of the peak firing rate.

The performance of pure and conjunctive neurons encoding a 2D stimulus is shown as a function of decoding time (T) and the number of cells available for decoding (N), for a different kind of scaling of peak firing-rates of conjunctive cells than the scaling used in **Fig. 3**. In 2D, we normalized the tuning curves such that the expected Fisher information at the limit of large N and T is equal for the two populations, but the mean-firing rate of pure and conjunctive cells is no longer the same. In this scaling the peak firing rate of conjunctive cells is chosen so that conjunctive cells fire on average fewer spikes than pure cells (see Methods), because as we discussed in the main text, when both populations have the same mean firing rate, the Fisher information of conjunctive cells is higher than that of the pure cells. Importantly, one can still observe the absolute advantage of either pure or conjunctive cells when N and or T are relatively small, respectively – even though their performance becomes exactly the same at large N and T, as expected from this scaling of their firing rates.

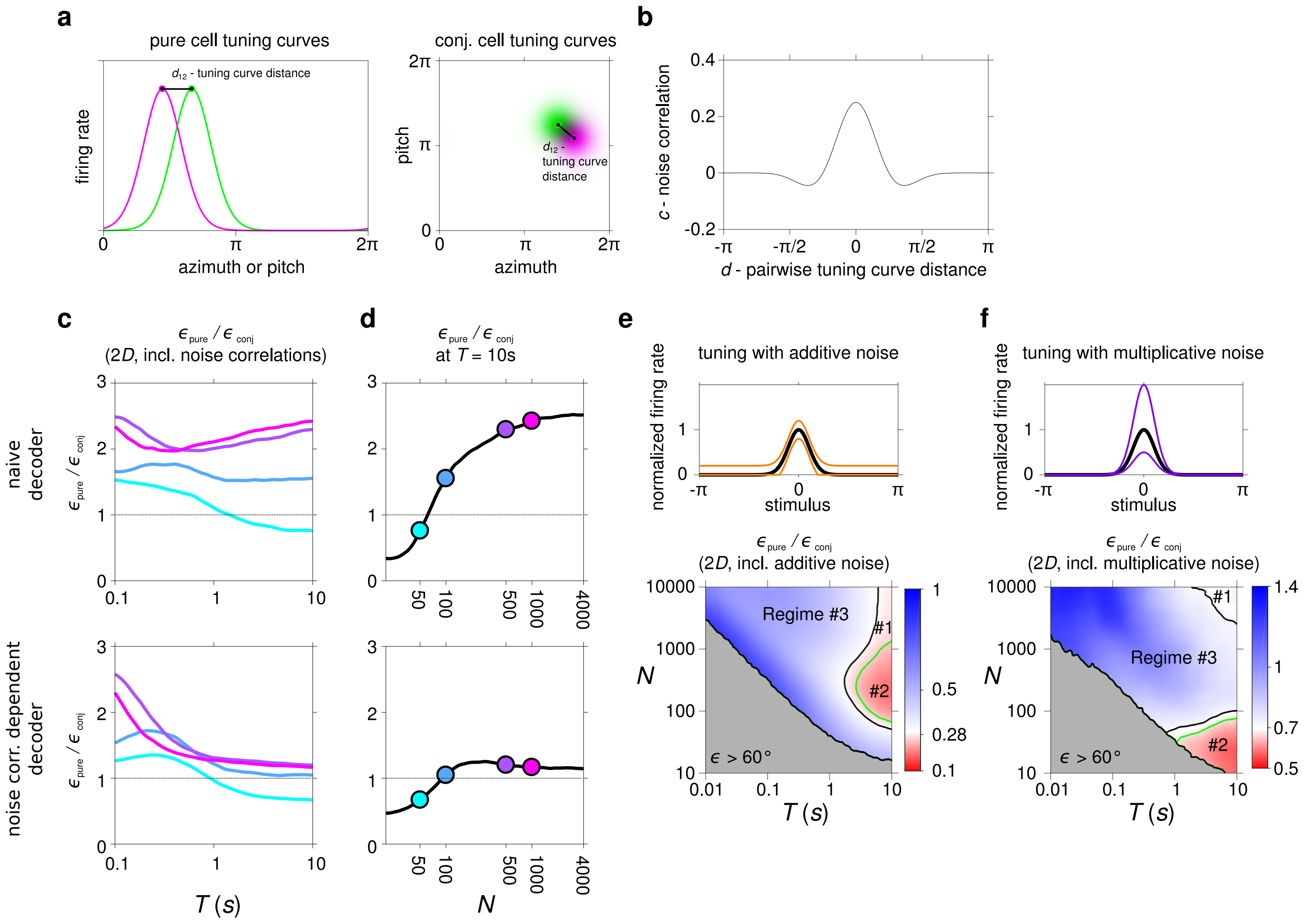

Supplementary Figure 4. Relative decoding performance of pure versus conjunctive cells in the presence of noise correlations and shared noise.

(**a-d**) We adopt here the noise correlation structure found in the head-direction system of rodents by Peyrache et al.[25], and show the relative performance of pure and conjunctive neurons encoding a 2D stimulus, as a function of decoding time (T) and number of cells available for decoding (N), in the presence of these noise correlations. (**a,b**) The noise correlation between every pair of neurons depends on their pairwise tuning curve distance d (see Methods), as illustrated in **a** for pure cells (left) and conjunctive cells (right). The distance is then transformed into a correlation value using the function illustrated in **b**. Cells with highly overlapping tuning curves are positively correlated, whereas cells with a small overlap are slightly negatively correlated. Given the correlation, spike counts are generated using a procedure detailed in the Methods. (**c,d**) Plot of the decoding error ratio $\epsilon_{\text{pure}}/\epsilon_{\text{conj}}$ of pure and conjunctive cells. Decoding was done using two different methods: a naive decoder that does not take into account the noise correlations (top) and a decoder that uses both the neurons' spike counts and the noise correlation structure to infer the stimulus (bottom). The dependence of the error ratio on N and T is rather similar to that found in the absence of noise correlations (compare to **Fig. 4c**). (**c**) The error ratio at short decoding times is larger than that at long times, indicating the relative advantage of conjunctive cells relative to pure cells at short times. As T increases the ratio saturates to a value that depends on N. (**d**) At long decoding times ($T = 10s$), decreasing N leads to smaller error ratio and eventually to an absolute advantage of pure cells, suggesting that at small N the conjunctive population suffers from a loss of coverage. (**e,f**) We plot the error ratio as a function of N and T in 2D using an alternative method

of introducing dependencies between cells. Here there is an overall additive (panel **e**) or multiplicative (panel **f**) random factor that modulates the tuning curves of all neurons in a sub-population (pure azimuth, pure pitch, conjunctive). This additive or multiplicative modulation was done prior to drawing the Poisson-distributed spike counts. The effect on the tuning curve is shown at the top panels. The bottom panels show that the error ratio exhibits the same qualitative behavior as in the case with independent spike counts. Because of this additional source of variability, the theoretical error ratio here is not $\sqrt{2}$. In the absence of an analytical calculation of the error ratio for large N and T for these noise models, we used the error ratio between pure and conjunctive cells found for the largest N, T values used in the simulation as the 'boundary' between the regimes.

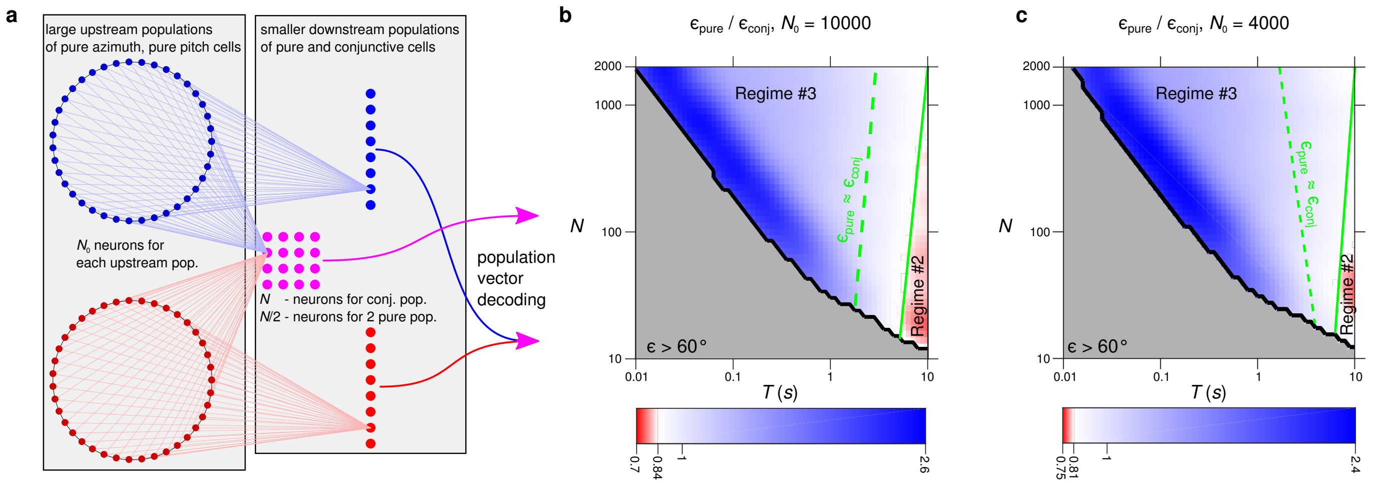

Supplementary Figure 5. Relative decoding performance of pure versus conjunctive cells in the presence of shared input.

(**a**) Schematic of a feed-forward two-layered model: the first layer (left) consists of a large number (N_0) of neurons tuned to either azimuth or pitch. Pure and conjunctive tuning in the second layer (right) is formed by pooling from the first layer. The inputs from the first to the second layer are scaled by excitatory synaptic weight profiles (see details in Methods). Responses in both stages are noisy, with Poisson statistics. Despite the fact that the Poisson noise for each neuron in each layer is independent, the spike counts are correlated both within and across the downstream subpopulations, due to the shared inputs. Decoding is done from the responses of neurons in the second layer: either from the pure azimuth and pure pitch populations (red, blue), or from the conjunctive population (magenta). (**b**) The decoding error ratio $\epsilon_{\rm pure}/\epsilon_{\rm conj}$ is plotted as a function of N and T, where N is the size of the downstream population and T is the integration time used to draw the spike counts of the upstream population. Here the size of each upstream population is $N_0 = 10,000$. Note, that when compared with the ratio at large N, T ($\epsilon_{\rm pure}/\epsilon_{\rm conj} = 0.84$), decoding is more accurate when using pure cell responses at small N (red region, analogous to Regime #2 in **Fig. 3**); whereas conjunctive cell responses yield more accurate decoding for small T (blue region, analogous to Regime #3). The regimes are well separated by the line $\log_{10}(N) = 7.45\log_{10}(T) - 4.15$ (solid green line, for which $\epsilon_{\rm pure}/\epsilon_{\rm conj} \approx 0.84$, the error ratio at large N, T). The dashed green line, for which $\epsilon_{\rm pure}/\epsilon_{\rm conj} \approx 1$, separates regions in $N - T$ space where either pure or conjunctive cells are absolutely better ($\epsilon_{\rm pure}/\epsilon_{\rm conj} > 1$ on the left, $\epsilon_{\rm pure}/\epsilon_{\rm conj} < 1$ on the right). (**c**) Same as panel **b** with smaller upstream populations ($N_0 = 4,000$), implying stronger noise correlations. The same qualitative behavior is observed: the error ratio at large N, T is 0.81, and the line that separates the regimes is now $\log_{10}(N) = 10.28\log_{10}(T) - 6.98$.

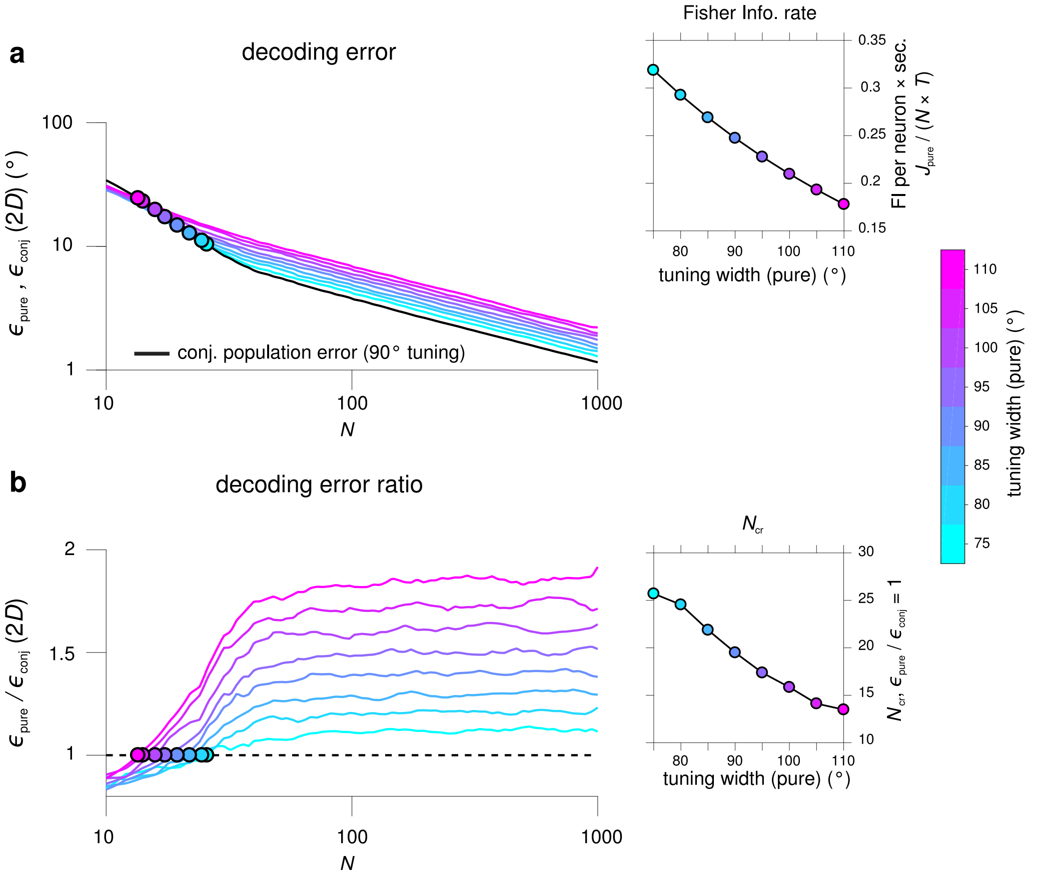

Supplementary Figure 6. Decoding accuracy of pure and conjunctive neurons depends on the relative tuning width of the two populations.

Here the two populations emitted the same number of spikes, on average. (**a**) The decoding error of a 2D stimulus is shown for conjunctive cells that have fixed tuning width (black) and pure cells for a range of tuning widths (see colorbar), as a function of the population size N, for fixed $T = 10s$. For small N, the pure cells outperform conjunctive cells. As N grows, all errors decrease – but the conjunctive cell error decreases faster, becoming smaller than that of pure cells (black line is below the colored lines for large N). Errors made by a pure population with broad tuning (magenta lines) are larger than those of a population with narrow tuning (cyan). This stems from the larger Fisher Information of pure cells with narrow tuning as compared to broad tuning, as shown in the inset. Note that the advantage of narrowly tuned over broadly tuned pure cells diminishes for small N where narrowly tuned cells suffer from poor coverage of the stimulus space. This can be seen from the limited range spanned by the colored lines (corresponding to decoding errors of pure cells with different widths) at small N. Inset shows the Fisher information rate of pure cells (the FI per neuron per second) as function of tuning-width. (**b**) The decoding error ratio, $\epsilon_{\mathrm{pure}}/\epsilon_{\mathrm{conj}}$, as a function of N, for different tuning widths of the pure cells. The ratio is equal to 1 at a critical value N_{cr} (black circles), which decreases as the tuning becomes broader (inset). Fisher Information increases as tuning becomes narrower (inset to **Supplementary Fig. 6a**), and therefore when the tuning of conjunctive cells is fixed, their advantage over a population of pure cells at large N diminishes as pure cells became more narrowly tuned (**Supplementary Fig. 6b**). Correspondingly, N_{cr} increased as pure cells became more narrowly tuned (inset to **Supplementary Fig. 6b**). Thus, both the difference in stimulus space coverage by pure and conjunctive cells, and the dependence of FI on the tuning width, contribute to the relative advantage of pure cells for small N and narrow tuning.

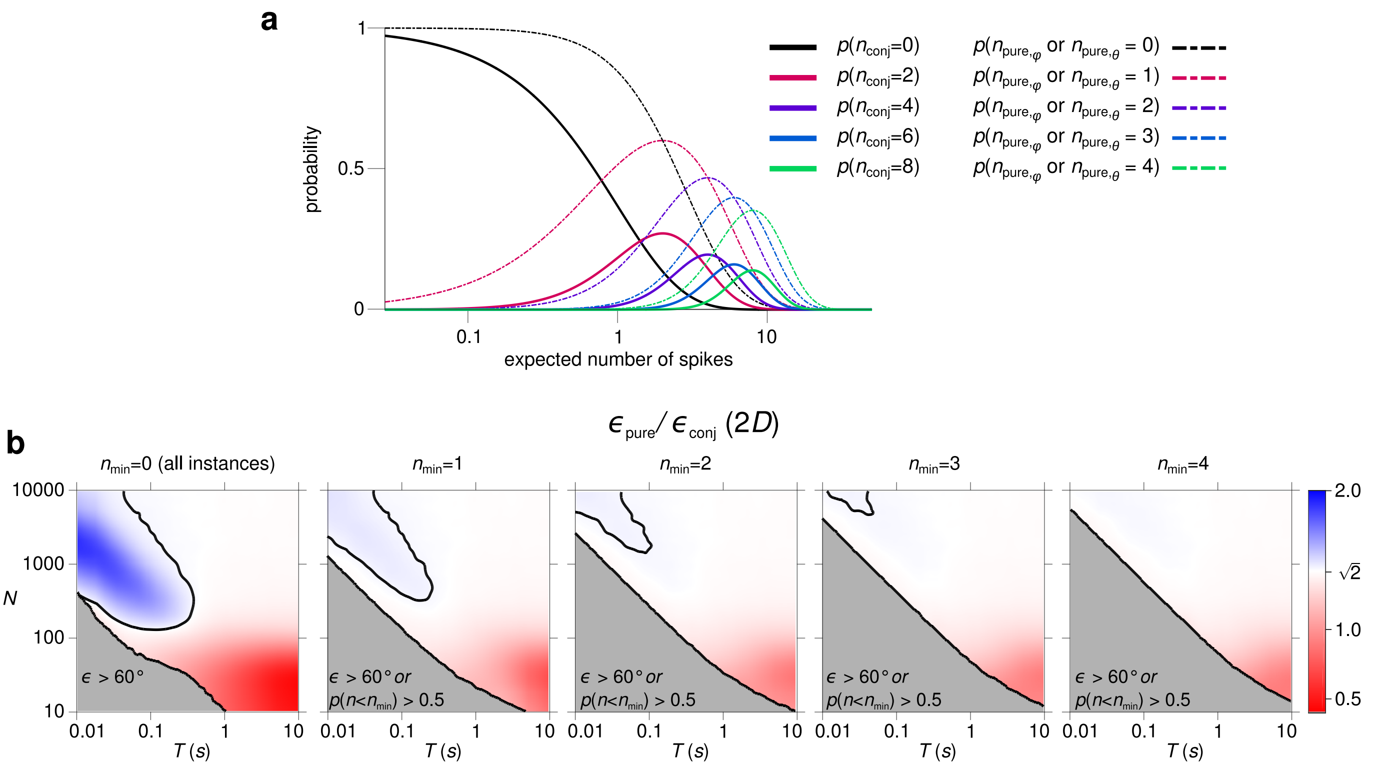

Supplementary Figure 7. Decoding from two pure subpopulations, each encoding a different stimulus dimension, is degraded when the estimate of the stimulus along one dimension fails.

(**a**) We computed the probabilities that one of the pure subpopulations will fire a very small number of spikes $n_{\rm min}$ (dashed lines); and that the conjunctive population will fire $2n_{\rm min}$ spikes (solid lines). These probabilities are shown as a function of the expected total number of spikes emitted by the entire population, which is the same for pure and conjunctive cells. When the expected number of spikes is 10-15, there is a large chance that the total spikes will be unevenly divided such that one of the pure subpopulations fires three spikes or less, leading to a very poor estimate of the stimulus along the direction corresponding to that subpopulation. (**b**) We computed the pure and conjunctive conditional errors – the decoding errors averaged after removing trials with few spikes as described in (**a**). The average number of spikes emitted by the pure and conjunctive populations remains equal after these trials are removed (see also Methods). The error ratio $\epsilon_{\rm pure}/\epsilon_{\rm conj}$ is shown after successively removing all trials where either of the pure subpopulations fired $n_{\rm min} = 0$ (taking into account all trials) and $n_{\rm min} = 1, 2, 3$ spikes. The black line, similar to **Fig. 3**, indicates an error ratio of $\sqrt{2}$. For $n_{\rm min} = 4$, Regime #3 where $\epsilon_{\rm pure}/\epsilon_{\rm conj} > \sqrt{2}$ is completely eliminated. This suggests that the advantage of conjunctive cells in this regime stems from instances with very poor estimation along a single dimension from the response of the pure population, because of insufficient number of spikes emitted by one of the pure subpopulations.

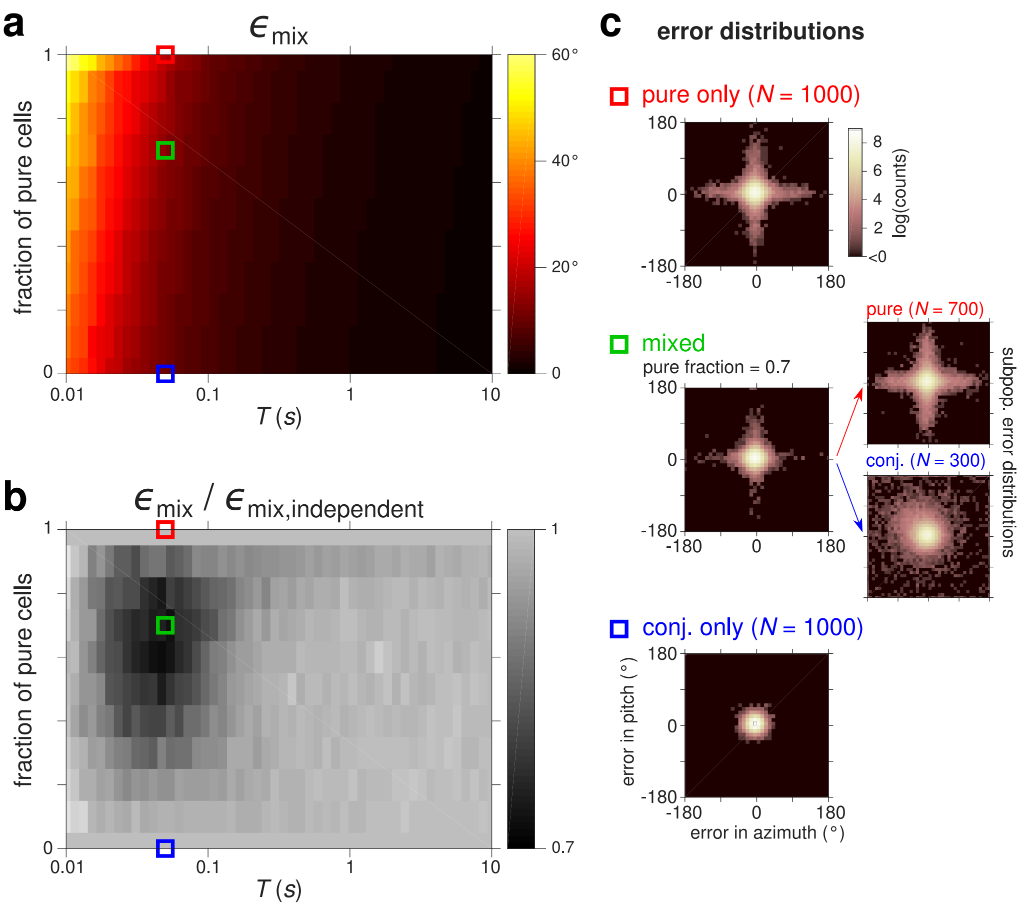

Supplementary Figure 8. A mixed population of pure and conjunctive cells can support more accurate decoding than expected from the two populations separately.

We computed the decoding error of a mixed population $\epsilon_{\rm mix}$ of total size $N = 1,000$, where the fraction of pure cells was varied between 0 (only conjunctive cells) to 1 (only pure cells). (**a**) As expected from **Fig. 3a**, where the error ratio favors encoding by conjunctive cells, the error of the mixed population is minimal for a conjunctive-only population, for all values of the decoding time T. (**b**) The error of the mixed population was then normalized by $\epsilon_{\rm mix,independent}$, the error that would be expected if the presence of two cell types in a single encoding population did not affect the decoding error (Methods). A ratio $\epsilon_{\rm mix}/\epsilon_{\rm mix,independent}$ below 1 (as observed here at short decoding times $< 0.1s$) indicates the presence of synergistic interactions between the subpopulations towards reducing the decoding error. (**c**) The error distribution is plotted for decoding time $T = 0.05s$, for a pure-only population (top), a mixed population composed of 70% pure and 30% conjunctive cells (middle), and a conjunctive-only population (bottom). The pure-only population suffers from poor decoding due to failed coincidence-detection along both stimulus-dimensions, evident from the cross-shaped error distribution (top) – which is ameliorated for the mixed population (middle) and conjunctive-only population (bottom). In the far-right column we plot the error distributions for the two subpopulations that comprise the mixed population – the pure cells (70%) and conjunctive cells (30%). When conjunctive cells comprise 30% of the mixed population the poor decoding due to failed coincident firing of pure cells is partially alleviated.

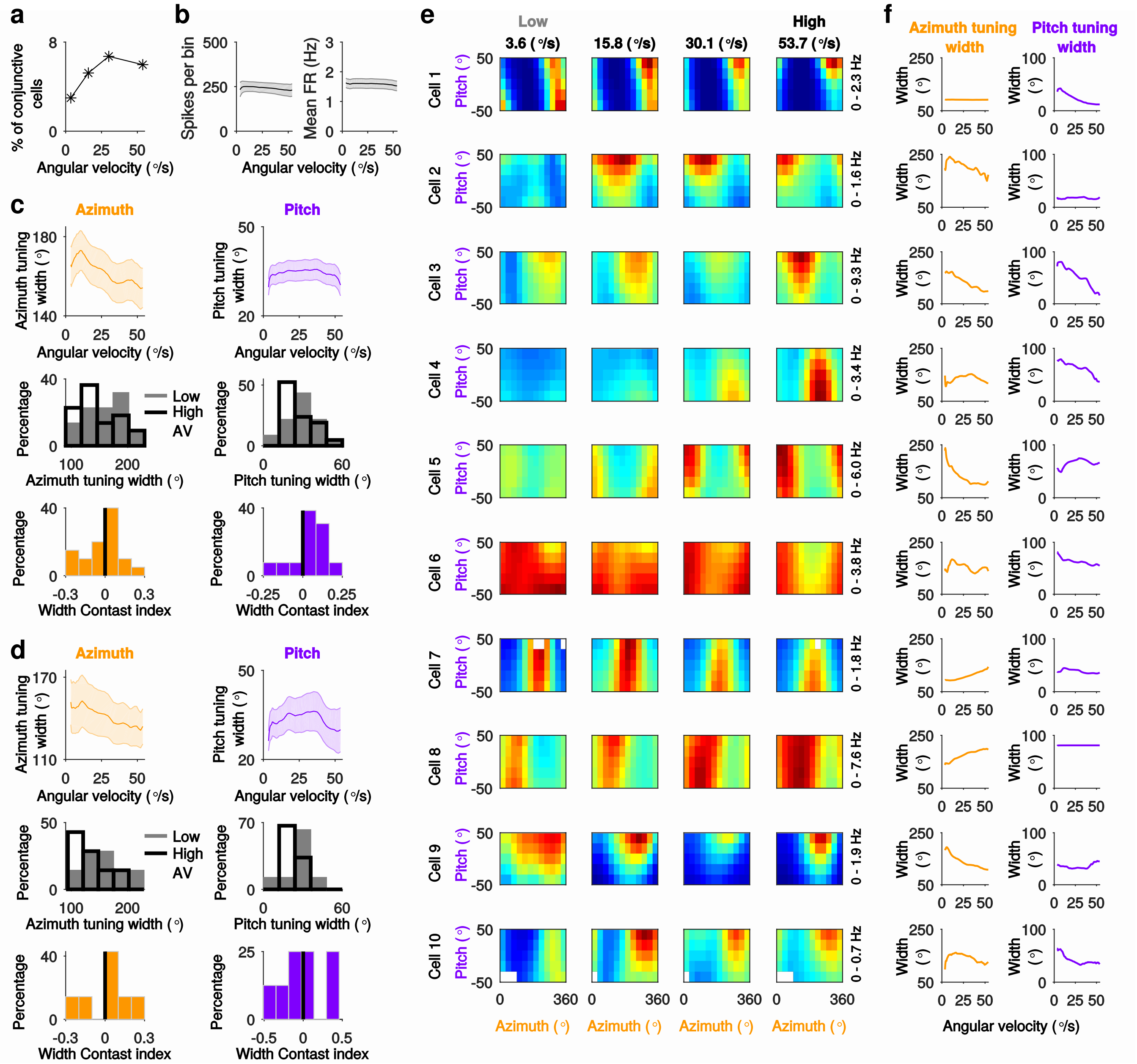

Supplementary Figure 9. Dynamic tuning in azimuth and pitch as a function of angular velocity.

We included in these analyses all the neurons with high spike-count (more than 400 spikes per session, $n = 134$ cells out of the original 266 cells). (**a**) Percentage of cells with significant conjunctive tuning as a function of combined (azimuth$\times$pitch) angular velocity (AV). Tuning was computed independently in 4 non-overlapping AV bins (each containing 25% of the behavioral data). (**b**) Median number of spikes (left) and mean firing-rate (FR, right) were computed in each angular velocity bin by moving it with increments of $1°s^{-1}$ per bin. (**c**) Tuning width in azimuth (left column) and in pitch (right column) for cells with significant conjunctive tuning in any of the 4 non-overlapping AV bins ($n = 24$ cells). Top, Tuning width as a function of angular velocity, averaged across cells. Middle, Distribution of tuning widths of individual cells, computed for the lowest AV bin (gray) versus the highest AV bin (black; same AV bins as in panel **a**). Bottom, width contrast-index, defined as $(W_L - W_H)/(W_L + W_H)$, where W_L and W_H are the tuning widths computed for the lowest and highest AV bins, respectively. Positive contrast index indicates a narrower tuning at fast AV. (**d**)

Same as in **c**, for cells with significant conjunctive tuning at high AV ($n = 8$ cells). (**e**,**f**) Azimuth and pitch tuning for example cells. Cell no. 1-8: all cells analyzed in **d**. Cells no. 9-10: additional examples. **e**, Shown are 2D rate-maps as a function of azimuth and pitch, computed separately for 4 non-overlapping angular-velocity bins (columns; the median angular velocity in each bin is shown above each column; same AV bins as in panel **a**). Color scale: zero (blue) to maximal firing-rate (red), values in Hz are indicated. **f**, Tuning width in azimuth (left) and pitch (right) as a function of angular velocity for each cell. In most cells, conjunctive tuning emerged at fast angular-velocities, as a result of progressive narrowing of tuning in azimuth, pitch, or both. Cells 9-10 also showed an emergence of conjunctive-tuning at high angular-velocity, but did not pass the formal significance criteria for conjunctive tuning.

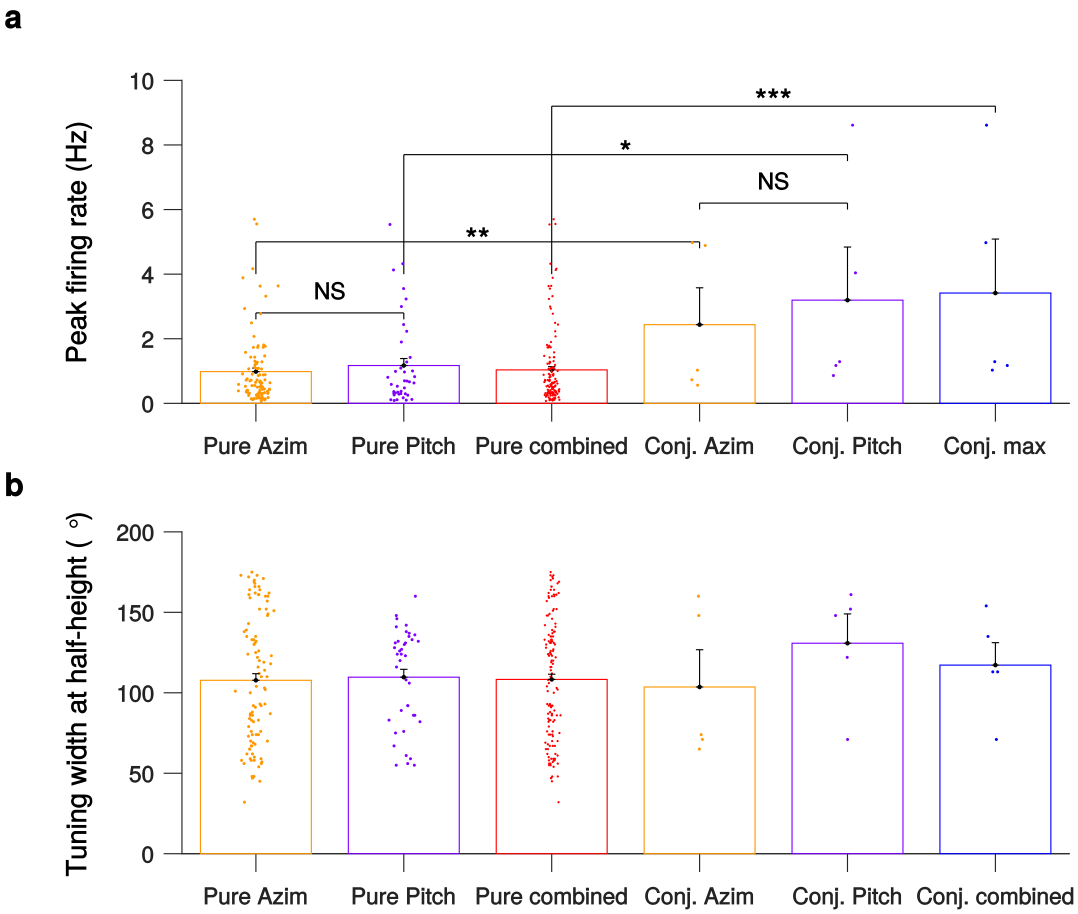

Supplementary Figure 10. Comparison of firing rates and tuning widths for pure versus conjunctive head-direction cells recorded in the bat dorsal presubiculum.

Average peak firing rate (**a**) and tuning width (**b**) for head-direction cells recorded in dorsal presubiculum of crawling bats reported in Finkelstein et al.[19]. We used 101 pure azimuth cells, 40 pure pitch cells, and 5 conjunctive cells for this analysis (see Methods). (**a**) Peak firing rates were very similar between pure azimuth and pure pitch cells, and therefore in the model we used the average peak firing rate of all pure cells ("pure combined") as a representative peak firing rate of the pure population. The conjunctive cells had significantly elevated peak-firing rate compared to pure cells. Note that this effect was significant both when the peak-firing rate of conjunctive cells was computed separately for azimuth and pitch dimensions (on the 1D marginal tuning curve for the respective dimension, "conj. azim", and "conj. pitch"), and when defined as the maximal peak-firing rate between the azimuth and pitch 1D tuning curves ("conj. max"). Error bars, mean ± s.e.m.; $^{*}P < 0.05$, $^{**}P < 0.01$, $^{***}P < 0.001$, using Student's t-test. (**b**) Tuning widths were very similar between recorded pure-azimuth and pure-pitch cells, and additionally there was no significant difference between the tuning width of pure and conjunctive cells – both when the tuning width was computed separately for the 1D tuning-curves, or taken as the average value across both dimensions ("combined"). Therefore we modeled the tuning curves with the same tuning width parameter for pure and conjunctive cells, in both azimuth and pitch dimensions.

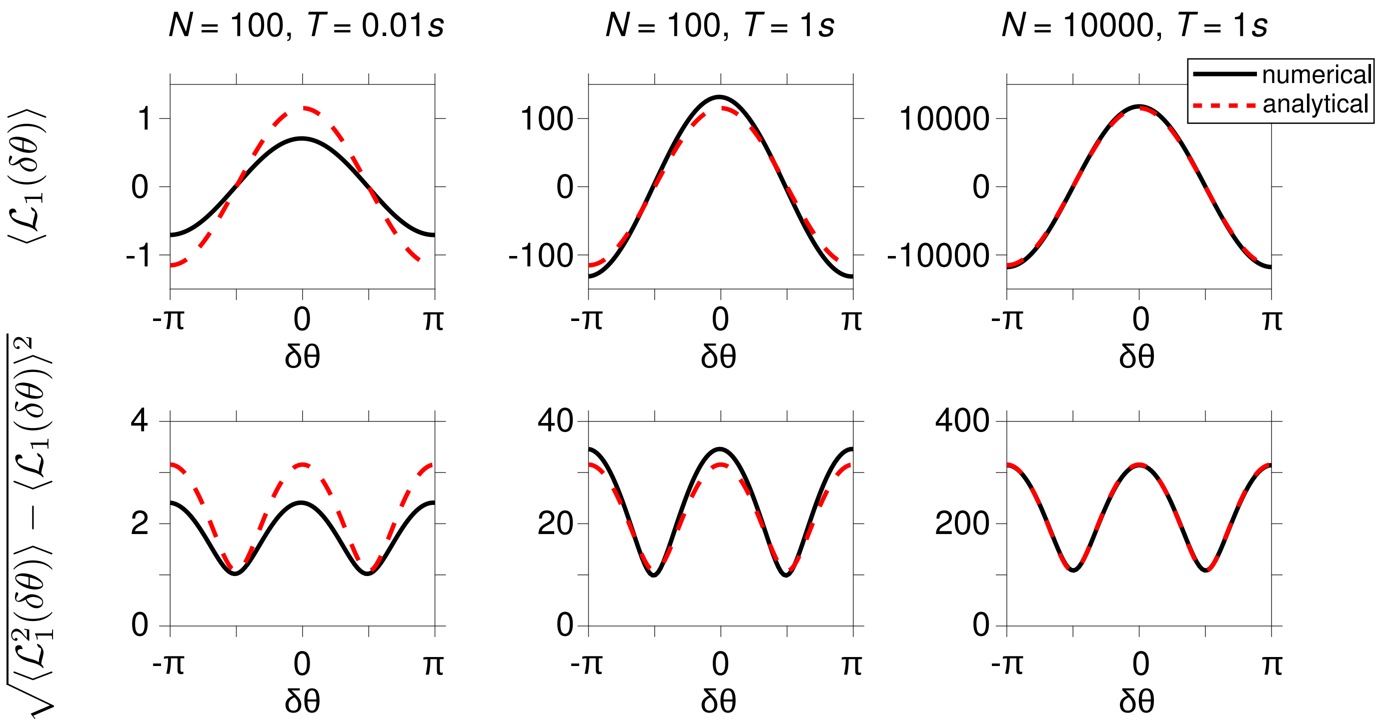

Supplementary Figure 11. Numerical and analytical evaluation of likelihood function moments.

Comparison of numerical (solid black) and analytical (dashed red) values of the first and second moments of the likelihood function $\mathcal{L}(\delta\theta)$ (top and bottom, respectively), for three sets of values of N and T. For small N and T, when the expectation value of the number of total spikes emmitted by the population is ~ 0.2, the approximation breaks down (left). When the number of spikes is increased either because of larger N or larger T, the approximation is improved. The approximation For N and T at which the ML decoding error is not saturated to the CR bound (when the average total number of spikes is 1-10, see **Fig. 4** in the main text) demonstrates that this approximation is accurate, validating our analysis.

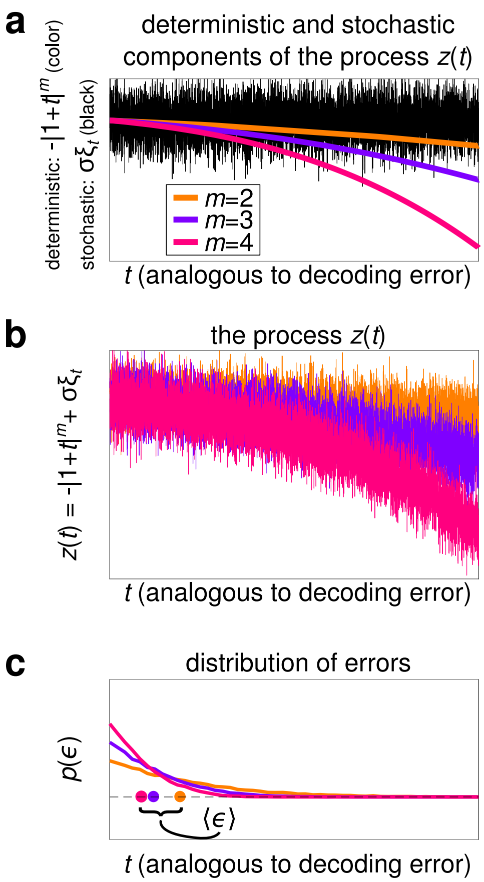

Supplementary Figure 12. Illustration of simplified decoding problem using likelihood function statistics.

(**a**) The statistics of the likelihood function $\mathcal{L}$ are assumed to be Gaussian. The mean is replaced by a deterministic algebraic decay shown in color for three values of m, and the fluctuations are replaced by Gaussian noise, shown in black. (**b**) The decoding error is approximated by finding the time t at which the sum of the deterministic and stochastic components are maximal. (**c**) The distribution of errors is shown for the three values of m (lines) along with the average (circles). The faster decay at larger m implies a smaller average error.

Supplementary Note 1

We study the scaling behavior of the Maximum Likelihood decoding error as a function of the population size N and the decoding time T. As $N, T \to \infty$, the average squared error saturates to the inverse Fisher Information, what is known as the Cramér-Rao bound.

To understand the scaling behavior of the error for finite N, T (i.e., away from the CR bound) we quantify fluctuations of the likelihood function used for decoding. If there were no fluctuations, the maximum of the likelihood function would be at the stimulus presented to the neuronal population. The random fluctuations "shift" the global maximum of the likelihood function away from the stimulus, leading to decoding errors.

The derivation is carried out in three steps:

First we average over the noise sources, namely the Poisson spike variability and the random preferred orientation of each neuron, to obtain expressions for the first and second order statistics of the likelihood function that is maximized for the decoded stimulus. Knowing these statistics allows us to write an effective simplified likelihood function that can be analyzed.

Second, for this effective likelihood function, we estimate the decoding error assuming a general algebraic form for the tuning curve.

Finally, using an effective form for any tuning curve shape (including the von Mises function we use throughout) we obtain an implicit equation for the ML decoding error, which is solved numerically. We conclude by comparing the result of our derivation to numerical simulations.

We emphasize that the derivation below is qualitative. As will be seen, it provides an explanation for two important observations we describe in the main text:

- In one dimension, for large N, the population size and decoding time control the error only through their product $N \times T$ (see **Supplementary Fig. 1b**).
- In two (or more) dimensions, for short decoding times, the error ratio favors conjunctive cells more than is expected from computing the FI (see blue region in **Figs. 3a,b** and **Fig. 4c**).

This derivation does not provide a quantitative account for the decoding error away from the CR bound. We think that the theory of extreme value statistics can be applied to obtain more refined estimates of interesting quantities. Doing so is beyond the scope of this paper and will be left for future work. We refer the reader to the book by Falk et al. (2004) that provides a thorough introduction to the theoretical tools one might use to study this problem. When relevant we point to key mathematical results we think may be useful.

First and second order statistics of the likelihood function

We start with a 1D stimulus s, and corresponding spike counts $n_i(s)$ drawn from a Poisson distribution with rate given by the von Mises tuning curve. The ML estimate of s is θ, and it is found by maximizing the likelihood function

$$\mathcal{L}(\theta, s) = \kappa \sum_{i=1}^{N} n_i(s) \cos(\theta - \theta_i) - TR \sum_{i=1}^{N} \exp\left[\kappa\left(\cos(\theta - \theta_i) - 1\right)\right]. \quad (1)$$

Here, κ is a parameter that controls the tuning width, θ_i is the preferred stimulus of neuron i, T is the decoding time, R is the peak firing rate and N is the size of the population.

The preferred stimuli are random and uniformly distributed between 0 and 2π. Throughout the derivation we assume that N is large enough such that for any function $g(\theta)$,

$$\sum_{i=1}^{N} g(\theta_i) \quad \rightarrow \quad \frac{N}{2\pi}\int_0^{2\pi} g(\theta')d\theta'. \tag{2}$$

We emphasize that the focus is on scenarios where the error deviates from the CR bound due to short decoding times T, *not* due to small N.

Define,

$$\begin{aligned} \mathcal{L}_1(\theta, s) &= \kappa \sum_{i=1}^{N} n_i(s)\cos(\theta-\theta_i), \\ \mathcal{L}_2(\theta) &= TR\sum_{i=1}^{N}\exp\left[\kappa\left(\cos(\theta-\theta_i)-1\right)\right]. \end{aligned} \tag{3}$$

Below we compute the averages $\langle\mathcal{L}_1\rangle$ and $\langle\mathcal{L}_1^2\rangle$. The assumption that sums can be replaced by integrals (Eq. 2) implies that $\langle\mathcal{L}_2\rangle$ is in fact a constant (it does not depend on the stimulus coordinate θ), so we drop it from our derivation and set $\mathcal{L} = \mathcal{L}_1$. Note that ignoring the term $\mathcal{L}_2$ is equivalent to using the population vector, rather than the maximum likelihood decoder. Averages are taken over multiple stimulus presentations and choices of the neurons' preferred stimuli.

The quantity $\langle\mathcal{L}_1\rangle$. This is simply the population vector. The average spike count is $\langle n_i(s)\rangle = RT\exp\left[\kappa\left(\cos(s-\theta_i)-1\right)\right]$. Defining $\delta\theta = \theta - s$ we write

$$\begin{aligned} \langle\mathcal{L}_1(\theta,s)\rangle &= \kappa e^{-\kappa}RT\sum_{i=1}^{N}\exp\left[\kappa\cos(s-\theta_i)\right]\cos(\theta-\theta_i) \\ &= \kappa e^{-\kappa}RT\sum_{i=1}^{N}\exp\left[\kappa\cos(s-\theta_i)\right]\left[\cos(\delta\theta)\cos(s-\theta_i)-\sin(\delta\theta)\sin(s-\theta_i)\right] \\ \langle\mathcal{L}_1(\delta\theta)\rangle &= \frac{1}{2\pi}\kappa e^{-\kappa}NRT\cos(\delta\theta)\frac{d}{d\kappa}\int_0^{2\pi}\exp\left(\kappa\cos\theta'\right)d\theta' \\ &= \kappa e^{-\kappa}I_1(\kappa)NRT\cos(\delta\theta) \\ &= J\cos(\delta\theta), \end{aligned} \tag{4}$$

where we have used standard identities for the modified Bessel function of the first kind $I_\nu(z)$, and where J is the Fisher information of a single population (see Eq. (7) in Methods).

The quantity $\langle\mathcal{L}_2\rangle$. For completeness, we computed $\langle\mathcal{L}_2\rangle$ which measures how evenly the neurons' tuning curves are spread in the stimulus space, and is thus independent of the stimulus coordinate θ and the stimulus itself s. A similar calculation gives,

$$\langle\mathcal{L}_2\rangle = e^{-\kappa}I_0(\kappa)NRT. \tag{5}$$

The quantity $\langle \mathcal{L}_1^2 \rangle$. Using the fact that for Poisson statistics the spike counts satisfy $\langle n_i^2 \rangle = \langle n_i \rangle^2 + \langle n_i \rangle$, the second moment of $\mathcal{L}_1$ is

$$\begin{aligned}\langle \mathcal{L}_1^2(\delta\theta) \rangle &= \langle \mathcal{L}_1(\delta\theta) \rangle^2 + NRT\kappa^2 e^{-\kappa} \left[\frac{1}{2} \left(I_0(\kappa) + I_2(\kappa) \right) \cos(2\delta\theta) + I_0(\kappa) \sin^2(\delta\theta) \right] \\ &= \langle \mathcal{L}_1(\delta\theta) \rangle^2 + \frac{\kappa \left[\frac{1}{2} \left(I_0(\kappa) + I_2(\kappa) \right) \cos(2\delta\theta) + I_0(\kappa) \sin^2(\delta\theta) \right]}{I_1(\kappa)} \times J. \end{aligned} \quad (6)$$

In the simulations, the decoder's performance is assessed by computing the estimated stimulus from instances of $\mathcal{L}$ in which the variability is generated by the Poisson statistics and random preferred stimuli are drawn from a uniform distribution. Possible interaction of these two noise sources and their non-Gaussian statistics makes analyzing how that variability affects the decoder's performance a difficult problem.

Instead, we study the performance of a hypothetical decoder which maximizes an effective likelihood function $\tilde{\mathcal{L}}$ that has Gaussian statistics with the same first and second moments as $\mathcal{L}$ that we computed above, i.e.,

$$\tilde{\mathcal{L}}(\delta\theta) = \langle \mathcal{L}_1(\delta\theta) \rangle + \sqrt{\langle \mathcal{L}_1^2(\delta\theta) \rangle - \langle \mathcal{L}_1(\delta\theta) \rangle^2} \xi(\delta\theta). \quad (7)$$

Here $\xi(\delta\theta)$ is a random Gaussian process with mean 0 and unit variance.

In addition to the variance shown in Eq. (6), the function $\mathcal{L}_1(\delta\theta)$ has non-zero auto-correlation: $\langle \mathcal{L}_1(\delta\theta) \mathcal{L}_1(\delta\theta + \Delta) \rangle \sim I_1(\kappa \cos(\Delta/2)) \neq 0$. This means that if the value of the likelihood function is large for some deviation $\delta\theta$ of the inferred stimulus from the actual one, it is likely large also for a "nearby" value of the deviation.

In the present work we aim to only obtain a qualitative understanding of the behavior of the error stemming from the fluctuations of the likelihood function. To do so we neglect the correlations of its fluctuations. It is in fact possible to include them by considering a correlated Gaussian process in $\tilde{\mathcal{L}}$, with correlations matched to those of the actual likelihood function $\mathcal{L}$. This however leads to expressions from which it is difficult to extract the error behavior. Specifically, doing so leads to a factor C_0 (see Eq. 13 below) which depends on ϵ. Assuming the autocorrelation is zero for $\Delta \neq 0$ leads to a constant C_0 which can be computed.

Substituting Eqs. (4,6) into Eq. (7) and dividing by J,

$$\begin{aligned}\tilde{\mathcal{L}}(\delta\theta) &= \cos(\delta\theta) + \sqrt{\frac{\kappa \left[\frac{1}{2} \left(I_0(\kappa) + I_2(\kappa) \right) \cos(2\delta\theta) + I_0(\kappa) \sin^2(\delta\theta) \right]}{I_1(\kappa)}} \times \frac{1}{\sqrt{J}} \times \xi(\delta\theta) \\ &= \cos(\delta\theta) + \frac{S(\kappa, \delta\theta)}{\sqrt{J}} \times \xi(\delta\theta), \end{aligned} \quad (8)$$

where we defined

$$S(\kappa, \delta\theta) = \sqrt{\frac{\kappa \left[\frac{1}{2} \left(I_0(\kappa) + I_2(\kappa) \right) \cos(2\delta\theta) + I_0(\kappa) \sin^2(\delta\theta) \right]}{I_1(\kappa)}} \quad (9)$$

On average, the effective likelihood function $\tilde{\mathcal{L}}$ has a maximum at $\delta\theta = 0$, so it correctly represents the fact that the ML decoder is unbiased. Instances of the random process ξ can shift the maximum away from 0 which we interpret as the errors that this effective decoder makes. In the next section we analyze the magnitude of these errors. In **Supplementary Fig. 11** we show a comparison of numerically computing the likelihood function $\mathcal{L}_1$ and the analytical estimate we use in the simulations (top row, Eq. 4; bottom row, Eq. 6). The formula we obtained breaks down when the entire population emits on average less than one spike (see **Supplementary Fig. 11**, left column, for which $N = 100$ and $T = 0.01s$). The approximation improves as the number of spikes grows (either as N is increased, or as T is increased), as can be seen in the middle and right columns of **Supplementary Fig. 11**.

The errors made by the effective Gaussian decoder with algebraic tuning

In the previous section we showed that the likelihood function maximized by the decoder can be written as a sum of a deterministic part and a stochastic part. The deterministic part is maximal for $\delta\theta = 0$ (i.e., the error in the absence of noise is 0). The stochastic part shifts the maximum away from $\delta\theta = 0$, meaning that in the presence of noise the error is non-zero. Now we want to use our knowledge of the mean and variance of this function to estimate the magnitude of errors by obtaining the characteristic distance of the peak of the likelihood function from $\delta\theta = 0$.

Analyzing the characteristic value of the error $|\delta\theta|$ at which the effective likelihood function (Eq. 8) has a maximum is difficult, so again we simplify. Consider the random process $z(t)$, starting at $t = 0$ and going only forward in time, such that

$$z(t) = -|1 + t|^m + \sigma\xi_t, \tag{10}$$

where ξ_t is a Gaussian random process with mean 0 and unit variance, and $m > 0$. Examples of this process are shown in **Supplementary Fig. 12a,b**.

This process is a simplified case of the Gaussian approximation we made to the decoding problem. In other words, $z(t)$, the position of the random process as a function of time, is analogous to $\tilde{\mathcal{L}}(\delta\theta)$, the value of the effective likelihood as a function of the decoding error. We refer to the decoding error coordinate $\delta\theta$ as the "time" t to emphasize the connection between the problem we are studying here and that of extremes of non-stationary random processes. In fact, in the Gaussian approximation, the likelihood function is a *nonstationary Gaussian process*. The mathematical properties of extreme values of this type of stochastic processes are discussed in chapter 10.3 of Falk et al. (2004).

In the case of the Gaussian approximation of the likelihood function (Eq. 8) the decoding error ϵ is equal to $\delta\theta$ for which the function $\tilde{\mathcal{L}}(\delta\theta)$ is maximal:

$$\tilde{\mathcal{L}}(\epsilon) \geq \tilde{\mathcal{L}}(\delta\theta), \tag{11}$$

for all $-\pi < \delta\theta \leq \pi$.

Note that we considered only a *one sided* process: $t \geq 0$. This choice ensures that the base of the deterministic term in Eq. (10) is always ≥ 1. This in turn guarantees that the deterministic contribution to $z(t)$ has monotonic dependence on m: if $m_1 > m_2$ then for all $t > 0$ we have that $-|1 + t|^{m_1} < -|1 + t|^{m_2}$ meaning that the decay of the deterministic part of $z(t)$ is faster for larger m. Had we considered also negative times, the m dependence of the deterministic term would change at $t = 0$.

Thus, in the simplified process (Eq. 10), we are interested in the time $t = \epsilon$ such that $z(\epsilon) > z(t)$ for all $t \geq 0$. We denote this time by ϵ, because the characteristic time by which the maximum of $z(t)$ is shifted from $t = 0$ will be interpreted as the decoding error, as in Eq. (11). **Supplementary Fig. 12** illustrates why ϵ depends on the exponent m: the deterministic part of the process $z(t)$ decreases faster for larger m, so the noise term is unable to "overcome" this decline such that the process reaches its maximum at later times, compared to the process $z(t)$ with a smaller value of m. The distribution of *errors*, i.e., times at which the process $z(t)$ reaches its maximum are shown in **Supplementary Fig. 12c**, along with the averages for three values of m.

The average of $z(t)$ (i.e., the deterministic part $-|1+t|^m$) decreases as time increases. The *maximum* of the stochastic part as a function of time, taken over realizations of the Gaussian noise, *increases* with time. This is true because the longer the process evolves, the more likely it is to have exceeded a given positive value in its history. The distribution

of extremes of *stationary* Gaussian processes (i.e., the case where σ in Eq. (10) is constant) has been computed analytically [see theorem 10.2.1 in Falk et al. (2004)].

Quantitatively computing the characteristic time at which $z(t)$ is maximal can thus be done by adding the deterministic part to the average over the distribution of extremes [Eq. (10.2) in Falk et al. (2004)]. It is also worth noting that this distribution might be useful in deriving an approximation of the *distribution* of errors (rather than the characteristic value) which is needed to obtain an expression for the *periodic* Cramér Rao bound [see Routtenberg & Tabrikian[58]].

Since we are only interested in the scaling of the characteristic time with the standard deviation σ and the exponent m, it is sufficient to inspect the probability that $z(\epsilon) > \langle z(t=0)\rangle = -1$, i.e., the probability that the process exceeds its initial value. For large enough t this probability is negligible meaning that there is essentially no chance that the decoder will make errors of the corresponding magnitude. This probability reads

$$\begin{aligned} p(z(\epsilon) > -1) &= \frac{1}{2\pi}\int_{-\infty}^{\infty} d\xi(\epsilon) \exp\left(-\frac{\xi^2(\epsilon)}{2}\right)\int_{-\infty}^{\xi(\epsilon)+\frac{1-(1+\epsilon)^m}{\sigma}} d\xi(0) \exp\left(-\frac{\xi^2(0)}{2}\right) \\ &= \frac{1}{2\sqrt{2\pi}}\int_{-\infty}^{\infty} d\xi(\epsilon) \exp\left(-\frac{\xi^2(\epsilon)}{2}\right)\left[1+\operatorname{erf}\left(\frac{\xi(\epsilon)}{\sqrt{2}}+\frac{1-(1+\epsilon)^m}{\sqrt{2}\sigma}\right)\right]. \end{aligned} \quad (12)$$

The integrand is a product of two terms: the first decays exponentially with $\xi(\epsilon)$; and the second increases monotonically but is bounded by 2 as the argument in the error function increases. Note that the Gaussian process $\xi(\epsilon)$ appears in the argument of the error function, and that increasing the error leads to *decreasing* the second term in the product.

Thus, as ϵ grows, the overlap of the region where both the $\exp\left(-\xi^2(\epsilon)/2\right)$ and $1+\operatorname{erf}(\cdot)$ terms are order 1 decreases. The maximal ϵ such that this overlap is non-negligible must satisfy

$$\frac{1-(1+\epsilon)^m}{\sigma} = -C_0, \quad (13)$$

with C_0 being a positive constant of order one. This is the maximal error ("time" in the language of the process z) for which there is a non-negligible probability of observing a global maximum in z.

For small ϵ we substitute $(1+\epsilon)^m \approx 1+m\epsilon$ into Eq. (13) giving,

$$\epsilon = C_0\sigma/m. \quad (14)$$

This scaling is in excellent agreement with numerical simulations (not shown). We will determine C_0 in the next section where we return to analyzing the approximate likelihood function we derived (Eq. 8), by requiring that the CR bound is saturated for small ϵ.

Estimating the errors of a maximum likelihood decoder

In the previous section we presented a simple scaling analysis of a random process with fixed algebraic drift and fixed variance (i.e., m and σ in Eq. 10 do not depend on t). The characteristic time $t=\epsilon$ at which the process $z(t)$ reaches its maximum is now interpreted as the decoding error, so $\epsilon = |\delta\theta|$ in Eq. (8). However, the quantities in the effective likelihood function we derived (Eq. 8), do depend on the stimulus coordinate (i.e., $\tilde{\mathcal{L}}$ is in fact a nonstationary process):

$$\begin{aligned} -|1+t|^m &\rightarrow \cos\epsilon \approx 1-\frac{\epsilon^2}{2}+\frac{\epsilon^4}{24} \\ \sigma &\rightarrow \frac{S(\kappa,\epsilon)}{\sqrt{J}}. \end{aligned} \quad (15)$$

We assume that the ϵ dependence of σ does not change the scaling relationship, and we introduce ϵ dependence to the scaling of the drift, i.e., $m = m(\epsilon)$, and

$$\epsilon = \frac{C_0 S(\kappa, \epsilon)}{m(\epsilon)\sqrt{J}}. \tag{16}$$

To determine an appropriate form of $m(\epsilon)$ and the value of C_0 consider the following two limits.

The limit $\epsilon \ll 1$: here we know from the Taylor expansion of cosine that $m(\epsilon \ll 1) = 2$; and that a very small error implies saturation to the CR bound, so $\epsilon = 1/\sqrt{J}$. Substituting these into Eq. (16) fixes $C_0 = 2/S(\kappa, 0)$.

The limit of large errors: since we are studying the decoding of a one dimensional angular variable, the error is bounded from *above* by $\pi/2$ regardless of how small J is. For $J = 1$ we assume the error is maximal, and neglect the dependence on κ. Under these assumptions Eq. (16) is satisfied if $m(\pi/2) = 2$. Including the κ dependence or choosing a different value of the FI (of order one) for which the error is maximal does not change the results qualitatively.

Between 0 and $\pi/2$, $m(\epsilon) < 2$ because the quartic term in the Taylor expansion of cosine is positive, so the effective drift is *slower* than $-\epsilon^2$. The simplest form of $m(\epsilon)$ that satisfies these requirements is

$$m(\epsilon) = 2 - \frac{\pi}{2}\epsilon + \epsilon^2. \tag{17}$$

Substituting Eq. (17) into Eq. (16) gives an implicit equation in ϵ for the scaling of the decoding error away from the CR bound:

$$\epsilon = \frac{S(\kappa, \epsilon)}{S(\kappa, 0)} \times \frac{2}{\left(2 - \frac{\pi}{2}\epsilon + \epsilon^2\right)\sqrt{J}}. \tag{18}$$

We solved Eq. (18) numerically, choosing a range of values for J. To allow comparison to numerical simulation of the ML decoder, we eliminate factors of order 1 by plotting the relative error $\epsilon/\epsilon_{\mathrm{CR}}$ (where $\epsilon_{\mathrm{CR}} = 1/\sqrt{J}$) which is expected to saturate to 1 from above for large J.

Plotted as a function of T, both the theoretical prediction and the numerical simulations show departure from the CR bound (dashed line at $\epsilon/\epsilon_{\mathrm{CR}} = 1$) that depends on the value of N, and both reach a saturation beyond which the error does not grow more with respect to the CR bound as time is decreased (**Supplementary Fig. 1**). There is no quantitative agreement in terms of the value of T at which the departure is observed, and no quantitative agreement for the maximal ratio. These differences could be expected since in our derivation we neglected factors of order 1 a number of times.

In the numerical solution of Eq. (18) and in the simulations of the ML decoder we used $\kappa = 9.1$ (corresponding to 45° tuning width), $R = 1$Hz and N between 100 and 10000. Importantly, when the relative error obtained from simulations is plotted as a function of $1/\epsilon_{\mathrm{CR}}$ instead of as a function of T, curves corresponding to different values of N collapse onto one another. This means that the error depends on N and T only through their product (which is proportional to J).

Decoding error of conjunctive cells

To obtain an estimate of the decoding error of conjunctive cells, we assume that the peak firing rate of conjunctive cells is normalized such that the average population firing rate of pure and conjunctive cells is equal (the normalization used

throughout the paper). Under this assumption, in two dimensions, the Fisher information of the pure cell population is equal to half that of the conjunctive cells.

Therefore, the estimate of the error of a ML decoder that uses conjunctive cell responses is obtained by solving Eq. (18) with $2J$ instead of J. One can expect then that the error ratio

$$\frac{\epsilon(J)}{\epsilon(2J)} \approx \frac{\epsilon_{\mathrm{pure}}}{\epsilon_{\mathrm{conj}}} \tag{19}$$

will exceed $\sqrt{2}$ for some values of J, because before convergence to the CR bound, multiplying the FI by 2 results in the error decreasing by a factor *greater* than $\sqrt{2}$.

Indeed this was the case when the ratio $\frac{\epsilon(J)}{\epsilon(2J)}$ was plotted (inset to **Fig. 4c**) for $N = 1000$ and $N = 2000$. Similarly to numerical simulations, the error ratio exceeds $\sqrt{2}$ for short decoding times, and converges to $\sqrt{2}$ for long decoding times, and the time at which the maximum ratio is attained depends on N. The theoretical estimate however fails to predict the fact that the maximal error ratio depends on N. This is accounted for by an analysis that uses a different approach, presented in the Methods section, and in **Supplementary Fig. 7**.

Supplementary References

Falk M, Hüsler J, Reiss R. 2004 Laws of small numbers: Extremes and rare events. Birkhauser Verlag. Basel, Switzerland.
