# Supplementary material for: Optimal dynamic coding by mixed-dimensionality neurons in the head-direction system of bats
Source: Nat Commun. 2018 Sep 4;9:3590. doi: 10.1038/s41467-018-05562-1 (PMC6123463; doi:10.1038/s41467-018-05562-1)
Supplement: Supplementary file 2 — Description of Additional Supplementary Files [file 41467_2018_5562_MOESM2_ESM.pdf]

Description of Additional Supplementary Files:

Supplementary Movie 1

Example of a typical natural flight outdoors of an individual Egyptian fruit bat, comprising both navigation and maneuvering. A typical nightly flight of an individual bat from its roosting cave to distal foraging sites (fruit trees). The commuting flight between the fruit trees is characterized by slowly-changing directional modulation (“navigation mode”), whereas foraging around the trees involves rapid changes of heading-direction in azimuth and pitch (“maneuvering mode”). The movie is sped-up 10× compared to real time (movie produced using desktop version of Google Earth Pro).
